# Epidemiology of Brucellosis Among Deer in China From 1978 to 2025: A Systematic Review and Meta‐Analysis

**DOI:** 10.1155/tbed/9727215

**Published:** 2026-05-20

**Authors:** Wen-Tao Xiang, Jia-Yu Yu, Qiu-Chi Jiang, Yi-Fan Zhang, Rui Liang, Ting Li, Wei Zheng, Jian-Ming Li, Fei Liu, Rui Du

**Affiliations:** ^1^ College of Animal Science and Technology, Jilin Agricultural University, Changchun, 130118, China, jlau.edu.cn; ^2^ Modern Agricultural College, Changchun Vocational and Technical University, Changchun, 130000, China; ^3^ College of Chinese Medicine Materials, Jilin Agricultural University, Changchun, 130118, China, jlau.edu.cn; ^4^ Department of Veterinary Medicine, College of Agriculture, Yanbian University, Yanji, 133002, China, ybu.edu.cn

**Keywords:** brucellosis, China, deer, meta-analysis, prevalence

## Abstract

Brucellosis is a major zoonosis affecting livestock production and public health. With the rapid expansion of sika and red deer farming in China, reports of deer brucellosis have increased. We searched CNKI, Wanfang Data, VIP, PubMed, and ScienceDirect and conducted a systematic review and meta‐analysis, including 47 studies published from January 1978 to September 2025 (54,699 samples). The pooled seroprevalence was 16.3% (95% CI: 10.9–22.4), with substantial heterogeneity. Subgroup analyses suggested an overall increase after 2000 and higher estimates in major deer‐farming areas of northern China. Seroprevalence tended to be higher in red deer than in sika deer, in adults than in subadults and juveniles, and in females than in males, and was also higher during summer and autumn. Method‐stratified subgroup meta‐regression did not detect statistically significant differences across diagnostic methods (*p* > 0.05); method‐based comparisons should be interpreted cautiously. In light of these findings, our study suggests strengthening deer surveillance systems, further standardizing diagnostic and reporting procedures, and implementing targeted control measures tailored to regional risk levels to reduce economic losses for farmers and lower infection in deer herds as well as the potential risk of zoonotic transmission.

## 1. Introduction

Brucellosis is a zoonotic bacterial disease caused by multiple species of the genus *Brucella* (*Brucella* spp.) and remains a widely distributed, persistent global public health concern. It remains a substantial challenge for both animal health and public health systems, especially in many low‐ and middle‐income countries, where decades of control and eradication programs have often failed to achieve durable control [1, 2]. According to Laine et al. [3], the annual number of human brucellosis cases worldwide is likely on the order of 1.6–2.1 million, rather than the oft‐cited 500,000. However, ongoing misdiagnosis, gaps in surveillance, and extensive underreporting suggest that the true burden is likely even higher [3–5]. Because brucellosis affects both human health and livestock production, major international agencies such as the World Health Organization (WHO), the World Organization for Animal Health (WOAH, formerly OIE), and the Food and Agriculture Organization of the United Nations (FAO) regard it as a priority zoonosis that requires sustained, coordinated control within a One Health framework [6].

Brucellosis mainly targets the reproductive system of affected animals, causing abortion, infertility, and a marked decline in overall productivity [7, 8]. In humans, the disease typically manifests as undulating fever, joint pain, and fatigue, and in some cases can progress to a chronic, multisystem condition [4, 9]. Humans are most often infected through direct contact with infected animals or their bodily fluids, especially during abortion or parturition, when bacterial shedding is at its peak. Infection may also be acquired by consuming raw or unpasteurized dairy products or meat that is undercooked and originates from infected animals [10, 11]. Most studies have focused on sheep, cattle, and pigs, which are typically associated with *B. melitensis*, *B. abortus*, and *B. suis*, respectively. However, more evidence shows that *Cervidae* (especially wild and farmed deer) can also be important reservoirs and sources of *Brucella* spp. transmission [12]. In Europe and North America, there have been reports of brucellosis in deer, and it has been shown to spread between species in some ecosystems [13, 14]. In sub‐Saharan Africa, wildlife brucellosis has also been seen in wild ungulates [15].

China is a major producer of farmed deer. Recently, rising demand for deer products, especially velvet antler, has driven rapid expansion of farming dominated by sika deer (*Cervus nippon*) and red deer (*Cervus elaphus*), with production concentrated in Jilin, Xinjiang, Liaoning, Heilongjiang, and Inner Mongolia [16, 17]. However, compared with cattle and small ruminants, deer have long been underrepresented in routine brucellosis surveillance and vaccination policies, creating gaps in risk assessment and control. Although brucellosis in deer herds has been reported in China, and some local surveys have recorded high positivity, the available data are geographically uneven and variable in quality, limiting robust national‐level inference. During data collection, we found that, in addition to the previously reported first human brucellosis outbreak in Guizhou linked to sika deer, a recent family cluster in Northeast China has also been reported in association with contact with sika deer. In both incidents, human brucellosis occurred after exposure to sika deer during slaughter or routine husbandry. These incidents highlight a clear public health risk and point to the need for tighter regulation of interprovincial deer movements and stronger biosecurity practices on farms [18, 19].

Field diagnosis of brucellosis in deer largely follows the same approaches used in cattle and sheep. In practice, it depends mainly on conventional serologic assays, including the Rose Bengal test (RBPT), the standard tube agglutination test (SAT), and various enzyme‐linked immunosorbent assay (ELISA) formats [20–22]. These assays are inexpensive and rapid, making them suitable for frontline use on farms; however, they are limited by the risk of false positives, operator subjectivity, and the lack of capacity to differentiate infected from vaccinated animals (DIVA) [23–25]. Molecular techniques, such as polymerase chain reaction (PCR) and loop‐mediated isothermal amplification (LAMP), have advanced quickly and can usefully complement serology for rapid confirmation and strain identification, but they require higher standards for laboratory conditions, operating procedures, and funding; consequently, standardized implementation and large‐scale deployment remain constrained [26–28]. Although China has issued the National Mid‐ and Long‐term Plan for the Prevention and Control of Animal Diseases (2012–2020) and the 5‐Year Action Plan for the Prevention and Control of Brucellosis in Animals (2022–2026), deer have not been systematically or comprehensively incorporated into nationwide mandatory surveillance or immunization systems, leaving gaps in species‐level management and control [29, 30]. Most published studies are one‐off cross‐sectional surveys, making it difficult to draw robust inferences about fluctuations and long‐term trends. In addition, farmed deer show pronounced regional clustering; information from southern provinces is limited but not absent—only single studies or small‐sample surveys have been reported in Linyi, Shandong (2012), Baoshan, Yunnan (2021), and Wuping, Fujian (2022) [31–33].

Taken together, studies on deer brucellosis in China exhibit substantial heterogeneity in diagnostic methods, sample representativeness, time spans, and geographic coverage, making it difficult to obtain reliable nationwide prevalence estimates. Although several local investigations have reported relatively high positivity in specific deer herds, the national evidence base remains patchy and varies considerably in methodological rigor. To fill this gap, we compiled all publicly available studies on deer brucellosis in China published from 1978 to 2025 and performed a systematic review and meta‐analysis. In this review, we set out to estimate the overall prevalence of brucellosis in farmed deer in China, to compare how often infection is detected by serologic tests, molecular assays, and bacterial isolation, and to explore how infection patterns vary across deer species and regions. We hope that these results will help shape surveillance systems that are specifically designed for deer, guide the choice and combination of diagnostic tools, improve the management of zoonotic risk, and contribute to more coherent One Health efforts in China.

## 2. Materials and Methods

### 2.1. Search Strategy

This systematic review and meta‐analysis was conducted and reported in accordance with the PRISMA 2020 Statement, and the completed PRISMA 2020 checklist is provided in Table S1 [34]. The search reporting followed the PRISMA‐S extension to ensure completeness and reproducibility [35]. We systematically searched five databases (CNKI, Wanfang Data, VIP (CQVIP), PubMed, and ScienceDirect) for primary studies published from January 1, 1978 to September 30, 2025, that reported the epidemiology of brucellosis in deer in China. Search strategies combined controlled vocabulary and free‐text terms and were adapted to each database. In PubMed, we conducted an advanced search combining MeSH terms and free‐text keywords for deer, brucellosis, and China, using (Deer) AND (Brucellosis) AND (China), with synonyms combined by OR within each concept. The full search string is provided in the Table S2. In ScienceDirect, we searched using the terms “brucellosis,” “deer,” and “seroprevalence.” For Wanfang Data, VIP (CQVIP), and CNKI, we searched using the Chinese equivalents of “deer” and “brucellosis” and performed backward citation tracking to minimize omissions.

We included peer‐reviewed primary studies conducted in China that (i) targeted deer (primarily sika deer and red deer) and (ii) reported original data on *Brucella* infection or seroprevalence (sample size and number positive) using serological, bacteriological, or molecular methods (e.g., RBPT, SAT, ELISA, PCR, LAMP, or bacterial isolation). We excluded reviews, conference abstracts, or case reports that lacked original data; non‐deer host studies (e.g., humans, cattle, sheep, pigs, and dogs); vaccine‐only, method‐validation, or experimental‐infection studies; and duplicate publications (retaining the most recent or most complete report). Eligibility criteria and study selection followed standard guidance to support clear PICO framing, comprehensive searching, and reproducible documentation.

### 2.2. Data Extraction and Quality Assessment

Data extraction was performed independently by two reviewers using a pilot‐tested, standardized form; discrepancies were resolved by a third reviewer. We extracted the following items: first author, publication year, sampling period, study location (province and region), deer species (red deer, sika deer, and others), diagnostic method (RBPT, SAT, ELISA, PCR, LAMP, or bacterial isolation), sample size, and number positive. When reported, host‐related variables (sex, age, and season) were also recorded. In addition, we extracted geo‐environmental variables (longitude, latitude, elevation, rainfall, temperature, humidity, and climate) when available in the article or its Supporting Information. Climate data were obtained from the China Meteorological Administration (CMA). For each sampling location, we used its latitude and longitude to match the corresponding—or nearest—meteorological station and extracted the relevant indicators (e.g., rainfall, humidity, and annual average temperature) for subsequent analyses. We did not contact the study authors for additional information because this review synthesized only published data.

Study quality was graded with a modified, GRADE‐based scheme, implemented as a 0–5‐point scale [36]. One point was awarded for each fully reported item: (i) whether random sampling was used, (ii) whether the sampling time was clearly reported, (iii) whether the sampling procedure was described in sufficient detail, (iv) whether the diagnostic method was clearly described, and (v) whether the study reported four or more relevant epidemiologic factors. Papers scoring 4–5 were considered high quality, 2–3 moderate, and 0–1 low; disagreements were resolved by consensus after discussion, as detailed in Table S3.

### 2.3. Statistical Analysis

All statistical analyses were conducted in R (version 4.4.3), using the {meta} and {metafor} packages for meta‐analytic workflows [37, 38]. The code for R in meta‐analysis is shown in Table S4. To stabilize the variance of proportions and approximate normality, we compared five transformations: raw proportions (PRAW), natural log (PLN), logit (PLOGIT), arcsine (PAS), and the Freeman–Tukey double arcsine (PFT) [39]. For each option, normality of the transformed study estimates was assessed using the Shapiro–Wilk test; the transformation closest to normality was used for the primary meta‐analysis (Table 1) [40]. Because some studies had zero events, the log and logit transformations resulted in NaN values. PFT and PAS can be calculated without continuity corrections; since PFT provides better variance stabilization at extreme proportions, we prioritized PFT.

**Table 1:** Normal distribution test for the normal rate and the different conversions of the normal rate.

Conversion form	*W*	*p*
PRAW	0.80701	2.286e − 06
PLN	NaN	NA
PLOGIT	NaN	NA
PAS	0.93956	0.01715
PFT	0.92788	0.006354

*Note:* NA, missing data; NaN, meaningless number; PAS, arcsine transformation; PFT, double‐arcsine transformation; PLN, logarithmic conversion; PLOGIT, logit transformation; PRAW, original rate.

Owing to marked heterogeneity, pooling was conducted under a random‐effects model. Between‐study heterogeneity was quantified with Cochran’s *Q* and *I* ^2^ statistics [41, 42]. Forest plots were produced for overall and subgroup syntheses; predefined subgroups included region, deer species, diagnostic method, sex, age, and season. Publication bias was examined using funnel plots and Egger’s tests (Table S5), complemented by trim‐and‐fill to explore the potential impact of missing studies [43, 44]. Robustness was evaluated via leave‐one‐out sensitivity analyses (iteratively omitting one study at a time). All statistical tests were two‐sided, and significance was defined as *p* < 0.05. Pooled prevalence estimates were reported with 95% confidence intervals and were back‐transformed to the original proportion scale for interpretation.

## 3. Results

### 3.1. Results and Quality Assessment of Included Literature

**Figure 1:**
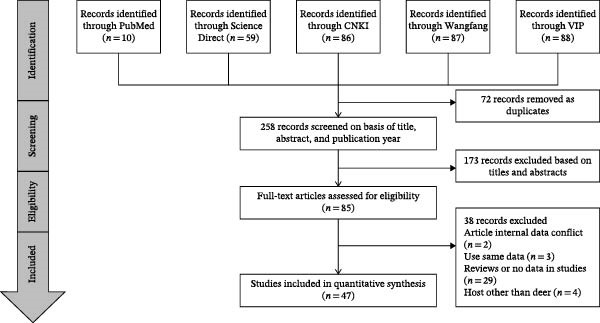
Flow diagram of eligible studies for searching and selecting.

In the initial database search, we identified 330 records on brucellosis in China using prespecified search terms. After applying the inclusion and exclusion criteria, 47 studies were included for meta‐analysis (Figure 1). Overall, the quality of the included studies varied. Most studies clearly reported the diagnostic methods and sampling time, whereas reporting was less complete regarding whether random sampling was used and whether the sampling procedure was described in sufficient detail. In addition, not all studies reported four or more relevant epidemiologic factors. Among the 47 studies, 15 were rated as high‐quality (scores 4–5), 32 were rated as moderate‐quality (scores 2–3), and none were rated as low‐quality (scores 0–1) (Table 2).

**Table 2:** Included studies of brucellosis in deer in China.

Reference ID	Sampling time	Province	Region	Variety	Detection methods	Season	Gender	Age	No. of tested	No. of positive	Prevalence (%)	Score	Quality level
Zhou et al. [45]	UN	Sichuan	Southwestern China	*Cervus nippon*	SAT	UN	UN	UN	92	20	21.74	2	Middle
Jia et al. [46]	1963	Heilongjiang	Northeastern China	*Cervus nippon*	UN	UN	UN	UN	397	294	74.06	3	Middle
Zhang and Qi [47]	1980	Henan	Central China	*Cervus elaphus*, *Cervus nippon*	PAT/SAT	Autumn	UN	UN	390	13	3.33	3	Middle
Jin [48]	1973	Jilin	Northeastern China	*Cervus nippon*	PAT/SAT	Winter	Male	UN	245	148	60.41	4	High
Ma [49]	1982	Qinghai	Northwestern China	*Cervus nippon*, *Cervus* elaphus	SAT	UN	UN	UN	173	31	17.92	4	High
Song et al. [50]	1979–1981	UN	UN	*Cervus nippon*	UN	Autumn	Male, female	Sub‐adult deer, adult deer, juvenile deer	523	59	11.28	3	Middle
Jin et al. [51]	1979–1986	Xinjiang	Northwestern China	*Cervus elaphus*	SAT/AGT	UN	UN	UN	47	4	8.51	4	High
Li et al. [52]	1986–1989	Jilin	Northeastern China	UN	PAT/SAT	UN	UN	UN	12,748	94	0.74	3	Middle
Bao et al. [53]	1991	Inner Mongolia	Northern China	*Cervus elaphus*	PAT/SAT	Spring	Male, female	Adult deer	27	12	44.44	4	High
Qu et al. [54]	1994	Jilin	Northeastern China	UN	PAT/SAT	Summer	UN	UN	2840	3	0.11	3	Middle
Chen et al. [55]	1995–1997	Jilin	Northeastern China	UN	ELISA/RBPT	UN	UN	Adult deer	300	42	14.00	3	Middle
Li et al. [56]	1991–1995	Jilin	Northeastern China	UN	UN	UN	UN	UN	2139	0	0.00	3	Middle
Jiang et al. [57]	1995–1998	Jilin	Northeastern China	UN	UN	UN	UN	Juvenile deer	504	24	4.76	2	Middle
Zhang et al. [58]	2001	Jilin	Northeastern China	*Cervus nippon*	RBPT/SAT	UN	UN	UN	728	20	2.75	4	High
Zhao et al. [59]	2001	Xinjiang	Northwestern China	UN	SAT	Autumn	Male, female	Adult deer	580	304	52.41	3	Middle
Chao [60]	2001	Qinghai	Northwestern China	*Cervus albirostris*, *Cervus nippon*, *Cervus elaphus*	SAT	Autumn	UN	Sub‐adult deer, adult deer	206	1	0.49	3	Middle
Mokhtar et al. [61]	2001	Xinjiang	Northwestern China	*Cervus elaphus*	PAT/RBPT/SAT	Summer	Male, female	Adult deer, juvenile deer	531	193	36.35	3	Middle
Cui et al. [62]	2002	Shanxi	Northern China	*Cervus elaphus*	ELISA/pathogen based method	Summer	Female	Adult deer	9	7	77.78	3	Middle
Zeng et al. [63]	2000	Xinjiang	Northwestern China	*Cervus elaphus*	PAT	Spring	UN	UN	173	7	4.05	4	High
Huang et al. [64]	2001	Xinjiang	Northwestern China	*Cervus elaphus*	SAT	Summer	Male, female	UN	3630	723	19.92	4	High
Li et al. [65]	UN	Jilin, Heilongjiang	Northeastern China	*Cervus nippon*	ELISA	UN	Male, female	UN	874	112	12.81	3	Middle
Zhang et al. [66]	1995–2004	Xinjiang	Northwestern China	*Cervus elaphus*	SAT	UN	UN	UN	6779	1968	29.03	3	Middle
Meng et al. [67]	UN	Xinjiang	Northwestern China	*Cervus elaphus*	RBPT/SAT	UN	UN	UN	300	47	15.67	2	Middle
Zhou and Fan [68]	2006	Xinjiang	Northwestern China	*Cervus elaphus*	RBPT	Summer	UN	UN	3100	206	6.65	3	Middle
Yan et al. [69]	2004–2005	Jilin, Heilongjiang, Liaoning, Xinjiang, Hebei	Northwestern China, Northern China	*Cervus elaphus*, *Cervus nippon*	RBPT/SAT	UN	Male, female	UN	3644	210	5.76	5	High
Li et al. [70]	2005	Jilin, Heilongjiang, Inner Mongolia	Northeastern China, Northern China	*Cervus nippon*	ELISA	UN	Male, female	Sub‐adult deer, adult deer, juvenile deer	1014	143	14.10	4	High
Dolkun et al. [71]	2005	Xinjiang	Northwestern China	*Cervus elaphus*	RBPT/SAT	Autumn	UN	UN	27	20	74.07	4	High
Yao and Yu [72]	UN	Liaoning	Northeastern China	*Cervus nippon*	RBPT	UN	UN	UN	541	144	26.62	2	Middle
Zhao and Zhang [73]	UN	Xinjiang	Northwestern China	*Cervus elaphus*	RBPT/PAT	UN	Male, female	Adult deer, juvenile deer	85	20	23.53	2	Middle
Yu et al. [74]	UN	Liaoning	Northeastern China	*Cervus nippon*	RBPT	UN	UN	UN	1055	213	20.19	2	Middle
Wang [31]	2009	Shandong	Eastern China	*Cervus nippon*	RBPT	Spring	UN	UN	1872	101	5.40	3	Middle
Mijiti and Dolkun [75]	UN	Xinjiang	Northwestern China	*Cervus elaphus*	RBPT/SAT	UN	UN	UN	61	48	78.69	3	Middle
Hasibat et al. [76]	2011	Xinjiang	Northwestern China	*Cervus elaphus*	RBPT	Autumn	UN	UN	72	11	15.28	3	Middle
Wu et al. [77]	UN	Xinjiang	Northwestern China	*Cervus elaphus*	PAT/SAT/PCR	UN	UN	UN	420	66	15.71	2	Middle
Wu [78]	UN	Jilin	Northeastern China	*Cervus nippon*	ELISA	UN	UN	UN	254	26	10.24	2	Middle
Liu et al. [27]	2011–2013	Jilin	Northeastern China	*Cervus nippon*	LAMP/PCR	Autumn	UN	UN	263	99	37.64	3	Middle
Liu et al. [26]	2016–2017	Jilin	Northeastern China	*Cervus nippon*	PCR	Summer, autumn	Male, female	UN	458	59	12.88	4	High
Shi et al. [79]	UN	Xinjiang	Northwestern China	*Cervus elaphus*	RBPT/SAT/RVFT	UN	UN	UN	674	163	24.18	2	Middle
Wang and Yan [80]	2019	Gansu	Northwestern China	*Cervus elaphus*	RBPT/SAT	UN	UN	UN	98	25	25.51	3	Middle
Wu et al. [81]	2009, 2011–2015	Xinjiang	Northwestern China	*Cervus elaphus*, *Capreolus pygargus*	ELISA	UN	UN	UN	72	2	2.78	5	High
Li et al. [32]	2017	Yunnan	Southwestern China	*Cervus nippon*	RBPT/SAT	UN	UN	UN	10	2	20.00	3	Middle
Zhong et al. [82]	UN	Fujian	Eastern China	*Cervus nippon*	RBPT	UN	UN	UN	150	0	0.00	2	Middle
Yuan et al. [83]	2021	Guizhou	Southwestern China	*Cervus nippon*	RBPT	Summer	UN	UN	56	15	26.79	3	Middle
Li [84]	2021–2022	Jilin	Northeastern China	*Cervus nippon*	SAT/RBPT/pathogen based method	Summer, autumn	Male, female	UN	783	41	5.24	5	High
Wang [85]	UN	Jilin	Northeastern China	*Cervus nippon*	GICA	UN	UN	UN	1240	72	5.81	3	Middle
Liao and Liu [33]	2022	Fujian	Eastern China	*Cervus nippon*	RBPT/ELISA	Spring	UN	UN	45	0	0.00	4	High
Tian et al. [86]	UN	Jilin	Northeastern China	*Cervus nippon*	ELISA	UN	UN	UN	4470	354	7.92	4	High

*Note*: Age: adult deer, (>2 years); sub‐adult deer, (1–2 years); juvenile deer (<1 year). Detection methods: PAT, plate agglutination test; RBPT, Rose Bengal test; SAT, standard tube agglutination test; ELISA, enzyme linked immunosorbent assay; AGT, anti‐human globulin test; GICA, colloidal gold immunochromatographic assay; RVFT, rapid vertical flow technology; PCR, polymerase chain reaction; LAMP, loop‐mediated isothermal amplification. Quality level: high (4–5); middle (2–3); low (0–1). Reference ID: references of the included articles in this meta‐analysis. Region: Northeastern China: Heilongjiang, Jilin, and Liaoning; Northwestern China: Gansu, Qinghai, and Xinjiang; Northern China: Hebei, Inner Mongolia, and Shanxi; Southwestern China: Guizhou, Sichuan, and Yunnan; Eastern China: Fujian and Shandong; Central China: Henan. Season: Spring: March to May; Summer: June to August; Autumn: September to November; Winter: December to February.

Abbreviation: UN, unclear.

### 3.2. Publication Bias and Sensitivity Analysis

The forest plot demonstrated very high between‐study heterogeneity (*I* ^2^ = 99.5%, p < 0.001) (Figure 2). The funnel plot suggested potential publication bias (Figure 3), which was supported by Egger’s test (*p* = 0.0280) (Figure 4). The trim‐and‐fill procedure imputed 20 studies with small effects and produced a lower pooled prevalence, indicating the presence of publication bias and highlighting the need for cautious interpretation (Figure 5). Sensitivity analyses indicated that excluding any single study through a leave‐one‐out analysis yielded conclusions consistent with the main analysis, further supporting the robustness of our meta‐analysis findings (Figure 6). The results of the meta‐analysis and publication bias of each subgroup are shown in Figures S1–S32.

**Figure 2:**
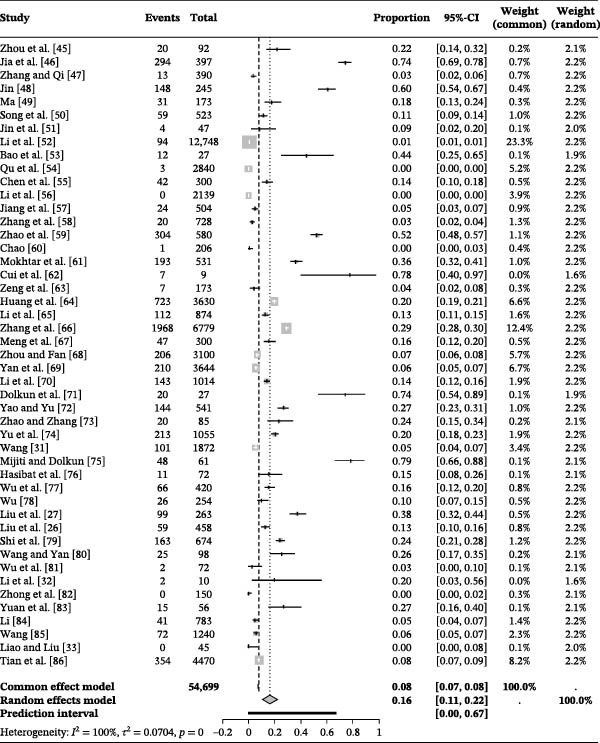
Forest plot of the prevalence of *Brucella* in deer amongst studies conducted in China. Pooled prevalence estimates with 95% confidence intervals (CIs) were calculated using random‐effects models. Between‐study heterogeneity was quantified using the *I* ^2^ statistic, and statistical significance was assessed using Cochran’s *Q* test (*p*‐value).

**Figure 3:**
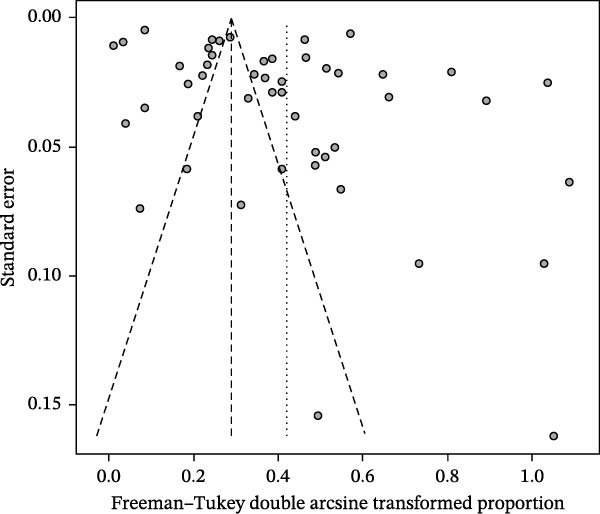
Funnel plot with pseudo 95% confidence interval limits for the examination of publication bias. In the absence of small‐study effects, studies are expected to scatter symmetrically around the pooled estimate; marked asymmetry may suggest potential publication bias or other small‐study effects.

**Figure 4:**
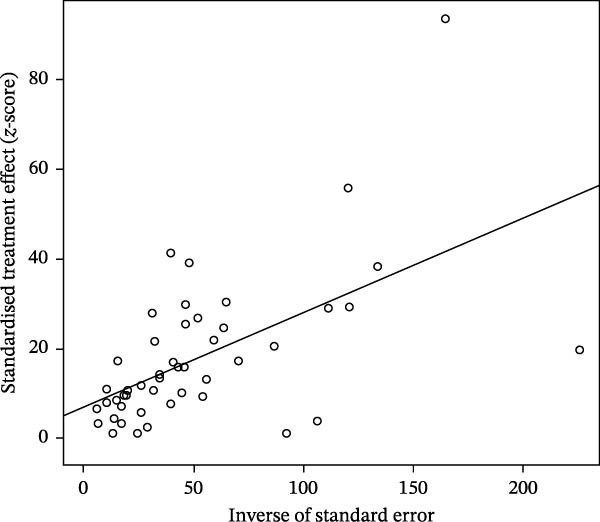
Egger’s test for publication bias.

**Figure 5:**
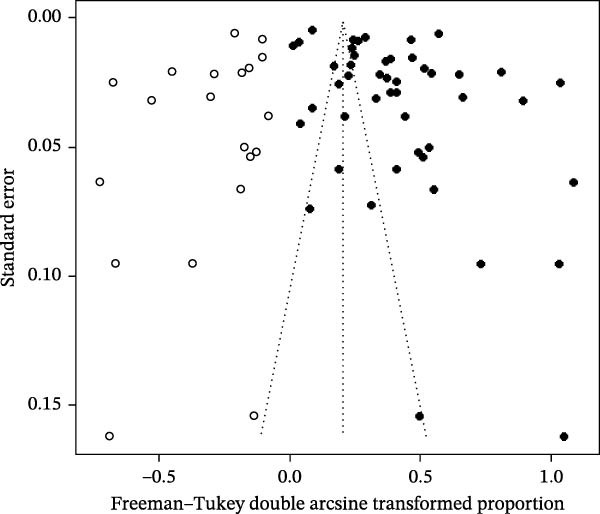
Trim‐and‐fill chart to detect research bias.

**Figure 6:**
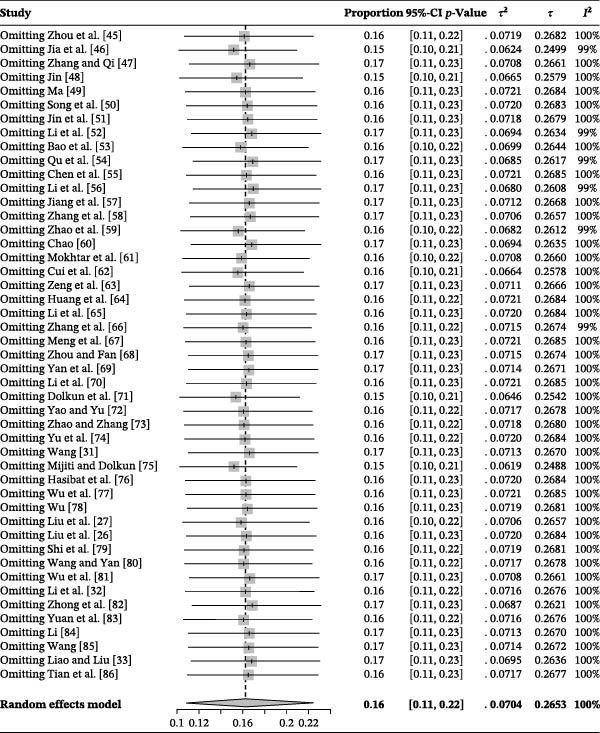
Sensitivity analysis. After one study at a time was removed, the remaining studies were recombined using a random‐effects model to verify the impact of a single study on the overall results.

### 3.3. Overall Seroprevalence of Deer Brucellosis in China Across Selected Periods

Figure 2 and Table 3 summarize findings from our systematic review and meta‐analysis of 47 studies comprising 54,699 clinical samples. The pooled seroprevalence of brucellosis in China’s deer populations was 16.3% (95% CI: 10.9–22.4). Notably, a few subgroup estimates were based on only one to two studies (as indicated in the “No. of Studies” column), resulting in wide confidence intervals; these estimates are presented for descriptive purposes only and should be interpreted with caution. By period, the estimates were as follows: 48.7% (95% CI: 14.2–84.0) before 1979; 6.7% (95% CI: 1.9–13.7) during 1980–1999; 18.5% (95% CI: 10.0–28.8) in 2000–2019; and 7.3% (95% CI: 0.0–27.2) since 2020. Notably, the post‐2020 estimate is based on limited evidence (*k* = 3; *n* = 884), and therefore, should be interpreted cautiously; it does not provide sufficient evidence to infer a sustained decline in prevalence after 2020 (Table 3).

**Table 3:** Aggregate prevalence of brucellosis in deer in China.

Subgroup	No. of studies	No. of tested	No. of positive	% (95% CI)	Heterogeneity			Univariate meta‐regression	
					*χ* ^2^	*p*‐Value	*I* ^2^ (%)	*p*‐Value	Coefficient (95% CI)
Region									
Northeastern China	23	33,650	2017	12.4 (6.5–20.0)	4146.03	0	99.5	—	—
Northwestern China	19	17,429	3877	21.1 (12.4–31.3)	1524.98	<0.0001	98.8	—	—
Northern China	4	482	62	30.7 (5.0–64.7)	60.71	<0.0001	95.1	—	—
Southwestern China	3	158	37	22.8 (16.2–30.0)	0.51	0.7739	0.0	—	—
Eastern China	3	2067	101	1.1 (0.0–5.9)	24.84	<0.0001	91.9	0.0394	−0.309 (−0.603– −0.015)
Central China	1	390	13	3.3 (1.8–5.4)	0.00	—	—	—	—
Study period									
1979 or before	3	743	456	48.7 (14.2–84.0)	136.40	<0.0001	98.5	0.0090	0.398 (0.100– 0.698)
1980–1999	11	19,590	268	6.7 (1.9–13.7)	484.76	<0.0001	97.9	—	—
2000–2019	19	23,266	4101	18.5 (10.0–28.8)	2565.74	0	99.3	—	—
2020 or late	3	884	56	7.3 (0.0–27.2)	26.66	<0.0001	92.5	—	—
Detection methods									
PAT	4	1199	299	18.8 (6.2–36.2)	144.04	<0.0001	97.9	—	—
RBPT	15	12,632	1320	13.2 (7.3–20.4)	763.49	<0.0001	98.2	—	—
SAT	21	31,256	3935	20.1 (10.6–31.6)	6350.55	0	99.7	—	—
Pathogen based methods	3	195	22	10.7 (0.6–27.8)	6.97	0.0307	71.3	—	—
ELISA	7	6993	686	13.7 (5.1–25.4)	76.59	<0.0001	92.2	—	—
PCR/LAMP	4	994	237	26.9 (14.5–41.4)	65.05	<0.0001	95.4	0.2622	0.140 (−0.105– 0.386)
GICA/RVFT	2	1914	235	13.7 (1.2–36.0)	127.83	<0.0001	99.2	—	—
Variety									
* Cervus nippon*	24	17,405	2077	13.6 (7.6–21.1)	1821.25	0	98.7	—	—
* Cervus elaphus*	21	17,687	3622	21.2 (12.6–31.3)	1409.44	<0.0001	98.6	0.1022	0.121 (−0.024– 0.267)
* Cervus albirostris*	1	68	0	0.0 (0.0–2.5)	0.00	—	—	—	—
* Capreolus pygargus*	1	16	0	0.0 (0.0–10.5)	0.00	—	—	—	—
Quality points									
2–3	32	39,163	4392	17.1 (10.5–25.0)	8391.04	0	99.6	0.6820	0.035 (−0.132– 0.202)
4–5	15	15,536	1774	14.5 (6.2–25.3)	939.09	<0.0001	98.5	—	—
Age									
Adult deer (>2 years)	9	2154	631	25.4 (10.8–43.3)	486.40	<0.0001	98.4	0.0224	0.244 (0.035– 0.453)
Juvenile deer (<1 year)	5	721	39	4.6 (2.8–6.8)	3.01	0.5554	0.0	—	—
Sub‐adult deer (1–2 years)	3	155	19	7.8 (0.1–23.0)	12.59	0.0018	84.1	—	—
Gender									
Male	12	8937	1124	17.3 (8.7–27.9)	716.08	<0.0001	98.5	—	—
Female	12	2623	786	27.9 (13.7–44.6)	639.43	<0.0001	98.3	0.2217	0.130 (−0.078– 0.337)
Season									
Spring	4	2117	120	8.4 (0.0–29.1)	32.32	<0.0001	90.7	0.3102	−0.165 (−0.483– 0.154)
Summer	8	11,146	1227	17.2 (5.0–34.1)	1514.86	<0.0001	99.5	—	—
Autumn	9	2175	512	19.0 (6.0–36.7)	642.60	<0.0001	98.8	—	—
Winter	1	245	148	60.4 (54.2–66.5)	0.00	—	—	—	—
Total	47	54,699	6166	16.3 (10.9–22.4)	9476.16	0	99.5	—	—

*Note*: Age: adult deer, (>2 years); sub‐adult deer, (1–2 years); juvenile deer (<1 year). Detection methods: PAT, plate agglutination test; RBPT, Rose Bengal test; SAT, standard tube agglutination test; ELISA, enzyme linked immunosorbent assay; AGT, anti‐human globulin test; GICA, colloidal gold immunochromatographic assay; RVFT, rapid vertical flow technology; PCR, polymerase chain reaction; LAMP, loop‐mediated isothermal amplification. Region: Northeastern China: Heilongjiang, Jilin, and Liaoning; Northwestern China: Gansu, Qinghai, and Xinjiang; Northern China: Hebei, Inner Mongolia, and Shanxi; Southwestern China: Guizhou, Sichuan, and Yunnan; Eastern China: Fujian and Shandong; Central China: Henan. Season: spring: March to May; summer: June to August; autumn: September to November; winter: December to February.

Abbreviation: CI: confidence interval.

### 3.4. Regional Seroprevalence Across Chinese Provinces and Administrative Regions

Our primary aim was to estimate seroprevalence and describe the spatial distribution of deer brucellosis in China. Over recent decades, however, deer populations have become unevenly distributed, and most studies and samples originate from Northeast China (*n* = 33,650) and Northwest China (*n* = 17,429). These two regions are the principal farming areas for sika deer and red deer in China. Reflecting this reality, our dataset is concentrated in these two regions; limited data from emerging farming areas in the central and southern provinces (notably sika deer operations) are included in supporting information. Therefore, for emerging farming regions such as Central, Eastern, and Southwestern China, subgroup pooled estimates based on smaller sample sizes or fewer included studies are subject to greater uncertainty (with wider confidence intervals) and should be interpreted with caution.

Seroprevalence varied by region (Table 3). Northern China (*n* = 482) had the highest pooled seroprevalence at 30.7% (95% CI: 5.0–64.7); Southwest China (*n* = 158), 22.8% (95% CI: 16.2–30.0); and Northwest China (*n* = 17,429, one of the principal regions for red deer farming), 21.1% (95% CI: 12.4–31.3). Northeast China (*n* = 33,650, one of the principal regions for sika deer farming) had a pooled estimate of 12.4% (95% CI: 6.5–20.0). Eastern China (*n* = 2067) and Central China (*n* = 390) were both below 5%, at 1.1% (95% CI: 0.0–5.9) and 3.3% (95% CI: 1.8–5.4), respectively.

**Figure 7:**
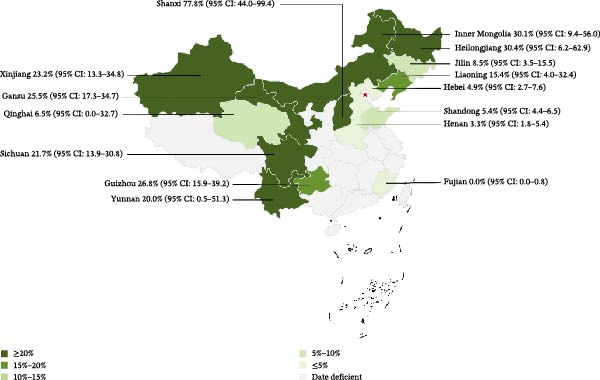
Map of *Brucella* in deer amongst studies conducted in China. Provinces are color‐coded by pooled seroprevalence range: ≥20%, 15%–20%, 10%–15%, 5%–10%, and ≤5%. “Data deficient” indicates provinces without eligible data for pooling. The color scale follows the legend shown on the map. Provincial estimates (with 95% CIs) are annotated and correspond to subgroup meta‐analyses (Table 4).

To describe the geographic coverage of the available evidence across provinces in China, we retained provincial subgroup estimates even when only a small number of studies were available for certain provinces. In this provincial stratification, Shanxi showed the highest point estimate of seroprevalence (77.8%; 95% CI: 44.0–99.4). However, this estimate was derived from a single study with a small sample size (*n* = 9) and a wide confidence interval, indicating considerable uncertainty; therefore, cautious interpretation is warranted when considering its relevance at the population level. Heilongjiang and Inner Mongolia followed, at 30.4% (95% CI: 6.2–62.9) and 30.1% (95% CI: 9.4–56.0), respectively. Provinces with 10.0%–30.0% included Guizhou, Gansu, Sichuan, Yunnan, and Liaoning. In Xinjiang (a traditional red deer region), the seroprevalence was 23.2% (95% CI: 13.3–34.8). In Jilin (one of China’s principal sika deer provinces), it was 8.5% (95% CI: 3.5–15.5). Provinces in the 0.0%–10.0% range included Qinghai, Shandong, Hebei, Henan, and Fujian; notably, both reports from Fujian recorded 0.0% seroprevalence in sika deer (Table 4 and Figure 7).

**Table 4:** Estimation of the prevalence of brucellosis in deer in the provinces.

Province	No. of studies	Region	No. of tested	No. of positive	Prevalence (%)	95% CI
Jilin	16	Northeastern China	29,741	1194	8.5	3.5–15.5
Liaoning	3	Northeastern China	2130	378	15.4	4.0–32.4
Heilongjiang	4	Northeastern China	1779	445	30.4	6.2–62.9
Xinjiang	16	Northwestern China	16,952	3820	23.2	13.3–34.8
Gansu	1	Northwestern China	98	25	25.5	17.3–34.7
Qinghai	2	Northwestern China	379	32	6.5	0.0–32.7
Hebei	1	Northern China	306	15	4.9	2.7–7.7
Inner Mongolia	2	Northern China	167	40	30.1	9.4–56.0
Shanxi	1	Northern China	9	7	77.8	44.0–99.4
Guizhou	1	Southwestern China	56	15	26.8	15.9–39.2
Sichuan	1	Southwestern China	92	20	21.7	13.9–30.8
Yunnan	1	Southwestern China	10	2	20.0	0.5–51.3
Fujian	2	Eastern China	195	0	0.0	0.0–0.8
Shandong	1	Eastern China	1872	101	5.4	4.4–6.8
Henan	1	Central China	390	13	3.3	1.8–5.4

### 3.5. Seroprevalence by Diagnostic Method

Most included studies used serological assays (Table 3). Twenty one studies employed the SAT, yielding a pooled seroprevalence of 20.1% (95% CI: 10.6–31.6); four studies used the plate agglutination test (PAT), yielding 18.8% (95% CI: 6.2–36.2); 15 studies used the RBPT, yielding 13.2% (95% CI: 7.3–20.4); seven studies used ELISA, yielding 13.7% (95% CI: 5.1–25.4); and two studies used colloidal gold‐based rapid tests (colloidal gold immunochromatographic assays [GICA] or rapid vertical flow tests [RVFTs]), obtaining 13.7% (95% CI: 1.2–36.0). In addition, four studies used molecular methods (PCR or LAMP), which showed a detection rate of 26.9% (95% CI: 14.5–41.4), exceeding that observed for conventional serology in these datasets. Three studies performed bacterial isolation, with an isolation rate of 10.7% (95% CI: 0.6–27.8). Interaction meta‐regression analyses did not detect significant interactions between diagnostic methods and the prespecified strata, including region, variety, study period, season, age, sex, and study quality (Table S6).

### 3.6. Host‐Related Risk Factors

Across host strata, age was the only statistically significant factor (Table 3). Adult deer (>2 years) showed the highest prevalence (25.4%; 95% CI: 10.8–43.3), followed by sub‐adult deer (1–2 years) (7.8%; 95% CI: 0.1–23.0) and juvenile deer (<1 year) (4.6%; 95% CI: 2.8–6.8), indicating an age‐related increase in infection risk (*p* < 0.05). When stratified by variety (Table 3), red deer had a higher point estimate than sika deer (21.2%; 95% CI: 12.6–31.3% vs. 13.6%; 95% CI: 7.6–21.1), but the difference was not statistically significant (*p* > 0.05). In sex‐stratified analyses (Table 3), females had a higher point estimate than males (27.9%; 95% CI: 13.7–44.6% vs. 17.3%; 95% CI: 8.7–27.9), yet this difference was also nonsignificant (*p* > 0.05).

### 3.7. Season‐Related Risk Factors

In season‐stratified analyses (Table 3), the winter farming period showed the highest prevalence at 60.4% (95% CI: 54.2–66.5), likely due to a single study (*k* = 1), and should be interpreted cautiously. In contrast, seroprevalence in spring, summer, and autumn was 8.4% (95% CI: 0.0–29.1), 17.2% (95% CI: 5.0–34.1), and 19.0% (95% CI: 6.0–36.7), showing a gradual increase from spring to autumn, although the differences across the three seasons were not statistically significant.

### 3.8. Geographic Risk Factors

Subgroup and univariable meta‐regression analyses showed clear spatial variation across longitude, latitude, elevation, and climate (Table 5).

**Table 5:** Geographical factor analysis of brucellosis in deer in China.

Geographical factor	No. of studies		No. of tested	No. of positive	% (95% CI)	Heterogeneity			Univariate meta‐regression	
						*χ* ^2^	*p*‐Value	*I* ^2^ (%)	*p*‐Value	Coefficient (95% CI)
Longitude										
80°–90°	7		8113	1464	26.3 (9.9–47.0)	873.60	<0.0001	99.3	0.2336	0.140 (−0.090– 0.370)
90°–120°	10		2886	207	15.5 (4.4–30.9)	165.04	<0.0001	94.5	—	—
120°–130°	9		4827	314	13.7 (4.5–26.7)	933.36	<0.0001	99.1	—	—
Latitude										
25°–40°	9		2859	195	12.9 (2.8–28.0)	139.49	<0.0001	94.3	—	—
40°–45°	14		12,570	1711	18.1 (9.1–29.3)	2469.43	0	99.5	—	—
45°–50°	3		397	79	30.3 (0.9–75.3)	82.82	<0.0001	97.6	0.3455	0.157 (−0.169– 0.484)
Altitude (m)										
0–250	8		6465	241	7.7 (2.5–15.4)	410.72	<0.0001	98.3	—	—
250–500	4		669	187	15.6 (0.2–46.2)	225.43	<0.0001	98.7	—	—
500–1000	8		7960	1480	38.6 (21.4–57.3)	871.06	<0.0001	99.2	—	—
1000–2500	6		732	77	10.8 (3.0–22.0)	83.96	<0.0001	94.0	0.0007	0.332 (0.139– 0.524)
Rainfall (mm)										
0–200	3		4741	1220	35.6 (18.3–55.1)	276.10	<0.0001	99.3	—	—
200–500	11		1898	310	25.3 (10.2–44.1)	566.42	<0.0001	98.2	—	—
500–1000	8		5721	207	8.0 (2.5–15.9)	374.59	<0.0001	98.1	0.0703	−0.203 (−0.423–0.017)
1000–2500	3		366	42	9.2 (0.0–29.8)	22.73	<0.0001	91.2	—	—
Humidity										
40%–60%	12		8694	1557	26.5 (13.1–42.5)	998.11	<0.0001	98.9	0.0584	0.193 (−0.007– 0.392)
60%–70%	10		4119	399	13.4 (5.2–24.5)	493.33	<0.0001	98.2	—	—
70%–80%	4		3013	29	6.2 (0.0–23.5)	95.79	<0.0001	96.9	—	—
Annual average temperature										
−5–5°C	3		296	83	36.5 (7.5–72.2)	30.92	<0.0001	93.5	0.1467	0.237 (−0.083– 0.557)
5–10°C	13		5307	338	13.0 (5.3–23.3)	968.45	<0.0001	98.8	—	—
10–25°C	10		10,223	1564	19.2 (6.9–35.4)	1112.02	<0.0001	99.2	—	—
Climate										
Temperate monsoon climate	8		6985	419	17.8 (2.7–41.0)	946.10	<0.0001	99.3	—	—
Temperate continental climate	9		8351	1517	25.4 (12.5–40.8)	879.82	<0.0001	99.1	0.2645	0.139 (−0.105– 0.384)
Subtropical monsoon climate	3		111	17	11.1 (0.0–38.4)	23.65	<0.0001	91.5	—	—
Plateau and mountain climate	2		379	32	6.5 (0.0–32.7)	47.66	<0.0001	97.9	—	—

Abbreviation: CI, confidence interval.

When stratified by longitude, the pooled seroprevalence rose from 13.7% (95% CI: 4.5–26.7) at 120–130°E to 15.5% (95% CI: 4.4–30.9) at 90–120°E and 26.3% (95% CI: 9.9–47.0) at 80–90°E—an apparent westward increase that did not reach statistical significance. With increasing latitude, estimates rose from 12.9% (95% CI: 2.8–28.0) at 25–40°N to 18.1% (95% CI: 9.1–29.3) at 40–45°N and 30.3% (95% CI: 0.9–75.3) at 45–50°N, although trend tests were not significant. By elevation, pooled seroprevalence differed significantly (*p* < 0.0001): 0–250 m, 7.7% (95% CI: 2.5–15.4); 250–500 m, 15.6% (95% CI: 0.2–46.2); 500–1000 m, 38.6% (95% CI: 21.4–57.3); and 1000–2500 m, 10.8% (95% CI: 3.0–22.0).

Regarding climate variables, prevalence tended to be higher in lower‐rainfall bands: 35.6% (95% CI: 18.3–55.1) at 0–200 mm, 25.3% (95% CI: 10.2–44.1) at 200–500 mm, 8.0% (95% CI: 2.5–15.9) at 500–1000 mm, and 9.2% (95% CI: 0.0–29.8) at 1000–2500 mm (*p* = 0.0703). For humidity, the 40%–60% band showed the highest estimate (26.5%; 95% CI: 13.1–42.5), dropping to 6.2% (95% CI: 0.0–23.5) at ≥70%. Mean temperatures of −5–5°C were associated with highest prevalence (36.5%; 95% CI: 7.5–72.2), compared with 5–10°C (13.0%; 95% CI: 5.3–23.3) and 10–25°C (19.2%; 95% CI: 6.9–35.4), and these associations were directional but not statistically conclusive. Across climate types, estimates were temperate continental climate, 25.4% (95% CI: 12.5–40.8); temperate monsoon climate, 17.8% (95% CI: 2.7–41.0); subtropical monsoon climate, 11.1% (95% CI: 0.0–38.4); and plateau and mountain climate, 6.5% (95% CI: 0.0–32.7).

## 4. Discussion

This study systematically reviewed research on brucellosis testing in deer in China from 1978 to 2025 and used meta‐analysis to comprehensively assess epidemiological characteristics, diagnostic methods, host factors, and eco‐environmental correlates such as season and geography. The results showed that the pooled seroprevalence of brucellosis in Chinese deer herds was 16.3% (95% CI: 10.9–22.4), with high between‐study heterogeneity (*I* ^2^ = 99.5%), indicating substantial differences across regions and study designs. Therefore, this estimate should be regarded as the overall average under a random‐effects model, and subgroup analyses are needed to describe how prevalence may vary across different risk factors.

This heterogeneity may arise from multiple sources. Differences across studies in diagnostic methods and testing workflows were not limited to the test type itself but also included variation in screening versus retesting procedures, positivity thresholds, and how positive results were counted and presented. Study design also varied in sampling strategies: in addition to routine large‐scale herd surveillance, some studies tested animals based on clinical signs, such as abortion in does or testicular swelling in stags, and others traced infected animals following human cases; in these situations, samples often came from herds with different underlying levels of disease risk, which may influence the overall estimates. Moreover, variation related to host factors and eco‐environmental conditions, including season and geography, may also exist. For example, herd management practices, animal movement history, cross‐species contact with domestic livestock such as cattle and sheep, and local environmental conditions may all contribute to differences in infection risk. Because the effective data provided by the primary studies were not consistent, these factors could not be jointly quantified in the models, and residual heterogeneity may remain. Future studies should further standardize study methods and reporting to improve comparability across studies.

A time‐series analysis shows that deer brucellosis in China has followed a “high‐low‐rebound” pattern over the past 50 years: 48.7% before 1979, 6.7% from 1980 to 1999, 18.5% from 2000 to 2019, and 7.3% since 2020. The early high levels probably reflected weaker quarantine systems and limited diagnostics, and county‐level investigations have observed insufficient regulation of animal movement and limited diagnostic capacity, which can increase transmission risk. The decline toward the late twentieth century coincided with stricter inspections, while the recent uptick aligns with industry expansion, more frequent cross‐regional movements, and lagging control capacity in some areas. In contrast, the pooled estimate after 2020 (7.3%) should be interpreted cautiously. Because evidence on deer brucellosis after 2020 is limited (*k* = 3; *n* = 884), this estimate alone is insufficient to infer a sustained decline in the prevalence of brucellosis in deer after 2020; the observed change may also be attributable to sparse post‐2020 data and publication lag. Although brucellosis control in livestock has been further strengthened following the implementation of the 2022–2026 5‐Year Action Plan for Brucellosis Prevention and Control in Livestock [30], which could theoretically reduce brucellosis transmission in deer, we were unable to match deer outcomes with contemporaneous livestock control intensity in the same locations and periods; therefore, we do not attribute the post‐2020 change in the pooled estimate to policy effects. Overall, the epidemiologic trends identified in this study are broadly consistent with the historically phased evolution of human brucellosis and animal (livestock) brucellosis in China, characterized by a higher burden before the 1980s, a relatively lower‐incidence period during the 1980s, and a subsequent rebound since the 2000s accompanied by some geographic expansion [87–89].

Additionally, fluctuations in compensation policies and economic incentives over time may impact trends in deer brucellosis seroprevalence. Because farmed deer have a high economic value and, in earlier periods, test‐and‐slaughter and compensation schemes for seropositive animals were often poorly implemented (with low compensation levels and delayed payments), some farmers tended to retain or sell suspect or confirmed animals, thereby prolonging the presence of infected animals within the herd and promoting the accumulation of subclinical infections and ongoing transmission. Tao et al. [18] investigated a human brucellosis outbreak linked to a sika deer farm in Guizhou. Their findings showed that, in the absence of an effective compensation mechanism, the farm owner chose to sell all of the sika deer to offset financial losses. The control measures that were actually implemented were largely limited to environmental disinfection and health education for workers, and no sanitary culling of seropositive animals was carried out. Similarly, Gong et al. [90] reported that in high‐incidence regions, integrated brucellosis control programs become difficult to sustain when government funding is limited, farmers lack the capacity to absorb financial losses, and compensation mechanisms fall short. Under these conditions, test‐and‐slaughter strategies become economically unsustainable, allowing infected animals to remain in herds and ultimately influencing overall prevalence. These findings indicate that the temporal trends in deer brucellosis are influenced not only by epidemiological processes and diagnostic methodologies but also by the development of policy frameworks and compensation systems that affect farmers’ behavior and, consequently, the long‐term efficacy of control measures.

Our findings indicate that brucellosis in farmed deer shows pronounced geographic variation and clear regional clustering across China. It is more common in the north than in the south and in the west than in the east. Regional subgroup analyses further indicate that the Northeast, Northwest, and Southwest have higher seroprevalence than Central and Eastern China. This pattern is corroborated by geographic stratification: seroprevalence increases from lower to higher latitudes and from east to west, which broadly aligns with the distribution of red deer farming in the Northwest and sika deer farming in the Northeast. Because deer farming is unevenly distributed across China and surveillance intensity varies by region, province‐specific evidence was sparse for several provinces. To maximize geographic coverage, we retained all eligible studies for the descriptive presentation of provincial estimates, including studies with small sample sizes. Nevertheless, the seemingly high estimate in Shanxi Province warrants cautious interpretation: it was derived from a single study with a very small sample size (*n* = 9), sampled from a deer farm experiencing a brucellosis outbreak and limited to aborting does, and the confidence interval was wide, reflecting substantial imprecision. Such single‐study, small‐sample estimates, particularly those arising from outbreak settings and clinically affected animals, are more susceptible to sampling variability and limited representativeness; therefore, this provincial estimate should not be overinterpreted as reflecting province‐level population risk without additional well‐designed surveys with adequate sample sizes and broader coverage.

Notably, the province‐level analyses show that Heilongjiang, Inner Mongolia, and Xinjiang each have pooled seroprevalence estimates exceeding 20%, indicating ongoing transmission in these regions. In contrast, Jilin and Liaoning—both of which have long‐established deer industries—show only moderate levels, likely reflecting more intensive production systems and stricter inspection practices. In contrast, Shandong and Fujian generally remain below 5%, reflecting low endemicity. These spatial patterns largely mirror the structure of the deer industry. In the major deer‐farming provinces of Jilin, Liaoning, Xinjiang, and Inner Mongolia, production is dominated by large, intensive operations with high stocking densities and frequent movement of animals across provincial borders. Active trade of breeding animals between farms, together with suboptimal biosecurity and inadequate isolation of newly introduced or suspect animals on some holdings, facilitates the silent circulation of *Brucella* within herds. Consistent with this, previous studies have reported that mixed‐species herds have significantly higher brucellosis seroprevalence than single‐species herds. For example, Jordanian cattle farms reported about twice the brucellosis positivity rate in mixed‐breeding conditions, while other studies documented even greater risks [91–93]. We observed similar findings through the subgroup analyses of geographic factors: elevation was the only significant geographic predictor (*p* = 0.0007), with the 500–1000 m band showing a seroprevalence of 38.6%, significantly higher than other elevation bands. Tracing the sampling sites indicates that studies in this band were largely concentrated in the major pastoral areas of Xinjiang and Inner Mongolia, where favorable climate–terrain conditions support extensive grasslands and local red deer farming is primarily domestication‐based grazing with supplemental feeding. During foraging, watering, and movement, deer herds overlap extensively with cattle and sheep at pastures and water points, increasing cross‐host contact and facilitating interspecies transmission.

This pattern is consistent with earlier findings. Oyetola et al. [94] showed that mixing different cattle groups at shared grazing sites and water points is a major risk factor for brucellosis transmission. Habimana et al. [95] similarly found that intergroup contact at common pastures and communal water sources can markedly increase the likelihood of spread. In pastoral production systems, the circulation of *Brucella melitensis* is typically closely linked to the status of brucellosis in sheep flocks; when interfaces such as shared grazing areas and watering points exist between sheep and other ruminants, the risk of cross‐host transmission may increase. Molecular evidence provides more direct clues to a “deer–sheep” link. Cao et al. [96] reported, based on WGS‐SNP analysis of isolates from Northwest China, that a deer‐derived isolate shared the same SNP genotype as a sheep‐derived isolate from Xinjiang, suggesting that deer and sheep may have a common source or a transmission link within the same regional production system. In addition, a typing study from Shaanxi offers supporting evidence: MLST results reported by An et al. [97], showed that *B. melitensis* (bv.1) was detected among deer‐derived isolates in their sample set, including sequence type ST8, indicating that infections in deer may involve species/lineages closely associated with brucellosis in small ruminants. Along the same lines, bovine‐associated lineages provide complementary evidence for a “deer–cattle” connection. In the identification and whole‐genome analysis of a deer‐derived *B. abortus* isolate (BJ1), Niu et al. [98] reported that BJ1 showed high homology to *B. abortus* 8416 isolated from a cleaner at a dairy farm in Baotou, Inner Mongolia, as well as to *B. abortus* MC and *B. abortus* BD isolated from aborted dairy cows in Mancheng County, Hebei, suggesting a close genetic relationship between deer infections and bovine‐associated lineages; however, the direction and specific pathways of transmission still require cross‐host coordinated surveillance and high‐resolution genomic tracing. Furthermore, Serrano et al. [99] noted that the persistence of brucellosis in red deer may be interconnected with brucellosis circulation in surrounding livestock (cattle, sheep, and goats); when brucellosis in livestock is controlled over the long term, brucellosis levels in red deer also tend to decline, implying synchronized changes between deer brucellosis and livestock brucellosis from a control‐practice perspective. Taken together, these molecular typing and genomic findings suggest that deer‐origin *Brucella* may be genetically linked to species or lineages circulating in cattle‐sheep production systems; therefore, geographic clustering and epidemiologic differences in deer brucellosis should be interpreted within the broader context of regional livestock brucellosis ecology and the farming environment.

In addition, Xinjiang and Inner Mongolia share the characteristics of a temperate continental climate, with low annual rainfall, relatively low humidity, long and cold winters, large diurnal temperature fluctuations, and frequent strong winds. These climatic features are consistent with our subgroup results for rainfall, humidity, temperature, and overall climate classification. This pattern corroborates the results of prior research conducted by Chen et al. [100] and Xu and Deng [101], which demonstrated that brucellosis is more common in the continental, arid, and semi‐arid grassland areas of northern China, closely linked to meteorological factors and grazing‐based production systems. Moreover, the WHO has clearly stated that *Brucella* can persist for prolonged periods in dust, feces, soil, water, and other environmental substrates, and that both humans and animals may become infected through contact with or inhalation of contaminated environments [102]. Although the available evidence does not allow us to infer a direct effect of climatic conditions on deer brucellosis, one possible explanation is that this climate–topography combination may, on the one hand, extend environmental persistence of contamination on soil, bedding, and pen surfaces; and, on the other hand, during dry–wet alternation, promote dust resuspension and runoff convergence to limited shared water sources, thereby increasing co‐exposure among sympatric animals. Conversely, southern deer farming tends to be smaller‐scale and more dispersed, with animals from single sources and less movement, resulting in lower risk. This north–south contrast is consistent with the findings of Sun et al. [103], who showed that brucellosis in China is predominantly concentrated in northern pastoral regions, whereas incidence in southern nonpastoral provinces is generally much lower and closely linked to differences in economic structure, livestock production systems, and meteorological conditions. WOAH explicitly classifies deer as susceptible to infection by *B. abortus*, *B. melitensis*, and *B. suis*. It imposes international trade requirements on deer similar to those for cattle and sheep, including herd disease‐free status or pre‐shipment quarantine; refer to the WOAH Terrestrial Manual and Terrestrial Code [104]. Accordingly, we recommend that deer farms in mixed husbandry areas follow these guidelines: avoid co‐grazing with cattle or sheep or sharing water sources; maintain separate zones for different species and enforce strict isolation during calving or abortion periods; and rigorously carry out quarantine and brucellosis testing for shipments between farms and provinces. Due to the spatial distribution of deer herds across the country, data from southern regions are relatively sparse. Overall, however, the persistence of brucellosis in deer appears to result from the combined influence of ecological suitability and differences in management practices.

From a diagnostic standpoint, although laboratory testing technologies are becoming more diverse, serology remains the most suitable choice for frontline field use for zoonotic diseases such as brucellosis [104]. In the studies included in this work, RBPT and SAT remain the most commonly used first‐line screening methods because of their low cost and simple operation; however, both also present issues, such as false positives and operator‐related problems and variability [22, 105]. In our meta‐analysis, the pooled estimates varied across diagnostic approaches (SAT 20.1%, RBPT 13.2%, ELISA 13.7%, PCR/LAMP detection rate 26.9%, and culture isolation rate 10.7%). However, univariable meta‐regression did not detect statistically significant differences between methods (*p* > 0.05), indicating that the available evidence is insufficient to infer method‐specific superiority or to attribute these differences to test performance rather than study design and reporting practices.

It is surprising that SAT was more positive than RBPT. RBPT is usually thought to be more sensitive and is used for initial screening. SAT is more specific and is often used for retesting or confirmation. Because of this, RBPT is expected to identify more positives than SAT. In our dataset, many studies used a sequential testing strategy in which animals were first screened with RBPT, and only RBPT‐positive cases were retested with SAT, but the published results counted only SAT‐positive animals as positive. As a result, RBPT‐reactive samples that did not meet the SAT criterion were excluded from the reported numerators, creating the misleading impression that SAT has a higher positivity rate than RBPT. Therefore, the difference observed in the pooled estimates between SAT and RBPT may be related to a two‐step testing algorithm (RBPT screening followed by SAT confirmation) and the practice of reporting only SAT‐positive results in the included studies. Additionally, variations in reagent sources, detection thresholds, and sample types across studies may further intensify statistical drift. Future reports should clearly separate RBPT screening and SAT retest positivity and explicitly specify whether a single‐test or combined algorithm was used, allowing for more precise meta‐analyses of sensitivity and specificity across serologic methods.

Compared with serological assays such as RBPT and SAT, the PCR/LAMP subgroup showed a higher positivity estimate. However, this finding should be interpreted in light of the application context: molecular assays such as PCR or LAMP typically require specialized instruments, reagents, and laboratory facilities and are therefore less frequently used in large‐scale routine surveillance in deer herds. Accordingly, in this meta‐analysis, the PCR/LAMP subgroup included relatively few studies and a small total sample size, which may increase the uncertainty of the pooled estimate. In addition, molecular results are sensitive to specimen type, target gene selection, nucleic acid extraction efficiency, and laboratory procedures, and may yield false‐negative results (e.g., low bacterial load) or false‐positive results (e.g., aerosol contamination or nonspecific amplification). Moreover, PCR/LAMP detects pathogen nucleic acids and more directly reflects whether the pathogen is currently present in the specimen, whereas serological markers more often reflect immune responses due to prior infection or vaccination; thus, positivity estimates derived from different testing modalities should not be treated as equivalent or compared directly. In this context, the choice between the two approaches should be guided by testing purpose: serology is generally more convenient for rapid, low‐cost population screening, whereas molecular assays can serve as an important complement when vaccination is used or when serological results may be affected by cross‐reactivity, helping with confirmation and adjudication. In addition, molecular methods support strain typing, molecular epidemiologic investigations, and source tracing, providing more direct evidence for targeted prevention and control.

To narrow the gap between laboratory testing capacity and the practical needs of farms, lateral‐flow rapid test strips have been developed, such as GICA and RVFT [20, 79, 106, 107]. These tools are portable, affordable, and easy for farmers or primary veterinarians to learn, making them suitable for initial screening and triage at deer farms. Meanwhile, nucleic‐acid and immunologic assays have advanced rapidly in recent years: recombinase polymerase amplification (RPA)/LAMP–CRISPR, droplet digital PCR (ddPCR), time‐resolved fluorescence lateral flow immunoassay (TRF‐LFIA), and aptamer–gold nanoparticle colorimetric biosensors (AuNP‐aptasensors) have collectively improved turnaround time, limits of detection (LoD), and tolerance to inhibitors relative to traditional methods [108–111]. Among these, ddPCR typically provides higher sensitivity and better inhibitor tolerance in complex, low‐copy matrices, and duplex ddPCR/quantitative real‐time PCR assays targeting vaccine‐specific loci have been developed for DIVA; however, systematic validation in deer remains lacking, and local evaluation is still needed [112, 113]. In addition, interferon‐gamma enzyme‐linked immunospot (ELISpot) testing in cattle and goats has shown high sensitivity and specificity and can detect infection earlier than SAT, indicating potential utility for early diagnosis [114]. Importantly, most of these advances are based on small cohorts of cattle, sheep, or humans; robust, deer‐specific cutoffs, antigen selection, and harmonized interpretation criteria are still lacking. We, therefore, recommend continuing to use accessible serologic tools for routine surveillance while prioritizing context‐specific validation in target deer species and against locally circulating field or vaccine strains (sensitivity, specificity, LoD, repeatability, and cross‐reactivity).

Host‐factor analysis identified age as the only statistically significant variable (*p* = 0.0224). Adults (>2 years) showed the highest seroprevalence (25.4%), followed by subadults and juveniles. WOAH explicitly states that pregnant animals can shed large quantities of bacteria into the placenta, fetal fluids, and vaginal secretions during abortion or parturition. It also indicates that *B. abortus* can be shed in semen from male animals. This aligns precisely with our analysis findings: *Brucella* transmission may be associated with mating between adult animals and exposure during parturition [104]. This suggests that brucellosis exhibits characteristics of chronic cumulative infection, with infection risk increasing with age.

Although the host‐species differences did not reach statistical significance (*p* > 0.05), the seroprevalence in red deer (21.2%) was higher than that in sika deer (13.6%), which may be related to host differences. Previous studies have shown that, compared with cattle, deer exhibit species‐specific differences in immune responses and vaccine response protection [115, 116], suggesting that host differences should be taken seriously and that the epidemiological patterns of brucellosis in cattle and sheep should not simply be extrapolated to deer. Based on these findings, control and surveillance strategies should be tailored for deer. First, deer‐specific immunization schedules and vaccine guidelines need to be established. Second, serologic assays for deer should be validated separately for each species, and diagnostic cutoffs should be revisited, using molecular tests as an adjunct for interpreting equivocal or discordant results. Third, the intensity and design of surveillance, including sampling intervals and target groups, should be tailored to local husbandry systems and regional risk, with additional monitoring during antler harvest, breeding, and parturition seasons. Finally, quarantine and isolation protocols need to be strengthened for animals moving between farms, newly introduced stock, and herds that may be involved in mixed grazing or share pastures and water sources with other species.

Sex was also not statistically significant (*p* = 0.2217), yet females showed higher seroprevalence rates (27.9%) compared to males (17.3%), consistent with *Brucella*’s affinity for reproductive tissues and increased exposure during pregnancy or parturition. This also aligns with our observations during data collection: 18 studies in the epidemiological investigations reported abortions, dystocia, or stillbirths among female deer at the surveyed farms, and 12 of these provided explicit abortion data. Mokhtar et al. [61] found that all 38 female deer that had abortions tested positive for brucellosis, with the highest agglutination titer being 1:600. Bao et al. [53] reported that at the Hanshan Deer Farm in Inner Mongolia, all 11 aborting hinds tested positive for *Brucella*. Therefore, we recommend that large‐scale farming operations, household farmers, and frontline veterinarians enhance biosafety awareness and adopt appropriate protective measures when performing on‐farm animal breeding, parturition assistance, and clinical examination and screening [117].

Based on the seasonal risk‐factor analysis, although differences across seasons did not reach statistical significance, the positivity rate increased progressively from spring to autumn, with summer and autumn clearly higher than spring; this seasonal pattern is plausible both biologically and in terms of farm management practices. Biologically, deer typically breed in autumn, and if *Brucella* infection occurs, abortions are likely the following spring. As a pathogen that strongly targets the reproductive system, *Brucella* shedding is closely associated with abortion and parturition, with risk concentrated from late spring to summer; this is consistent with Beauvais et al. [118] and Samadi et al. [119]: during the birthing season, both *Brucella* positivity and transmission intensity increase, primarily due to heavy shedding in parturition‐associated excretions and the resulting environmental contamination. From a husbandry perspective, sika deer retain more wild temperament than cattle or sheep, and venipuncture often requires anesthesia; consequently, farmers commonly increase testing during the summer velvet‐harvest period and before autumn breeding [16]. Therefore, higher positivity in summer and autumn accords with on‐farm realities. By contrast, the apparent winter elevation mainly reflects sparse evidence (*k* = 1) and older sampling and is not population‐representative.

Overall, deer brucellosis in China shows a pronounced spatiotemporal distribution and has remained at a relatively high level of endemicity. This study focuses on farmed deer populations; during transport, mixed housing, and slaughter, farmed deer may act as bridge hosts, facilitating the transmission of *Brucella* between other livestock and humans and increasing zoonotic risk. Future control should adopt a region‐specific, risk‐based strategy: establish routine surveillance and early‐warning systems in northern priority areas; advance integrated diagnostics by combining molecular assays with serology to improve epidemiologic sensitivity; strictly regulate the cross‐regional movement of deer products; and strengthen quarantine for breeding stock, velvet antler, and slaughter processes. We recommend strengthening brucellosis control for farmed deer within the national major animal disease control framework by aligning disease surveillance and early warning, animal movement management, quarantine, management of test‐positive animals, and zoonotic biosecurity measures with the control principles applied to livestock such as cattle and sheep, while further developing brucellosis quarantine and prevention guidelines tailored to cervids. Meanwhile, we recommend strengthening the development and evaluation of brucellosis vaccines for cervids to provide an evidence base for future immunization strategies. Under the One Health framework, coordinated cross‐species surveillance should be implemented to prevent deer‐derived strains from spreading to other livestock and humans.

Additionally, this study has several limitations: high between‐study heterogeneity caused by differences in diagnostic methods and reporting standards; limited sample size in some regions, which restricts geographic representativeness; few molecular studies, reducing the stability of method comparisons; and incomplete reporting of key host variables (sex, age, and season) in many primary studies, impacting the depth of multivariable analyses. Future research should develop standardized surveillance and data‐sharing platforms, specify diagnostic procedures, and expand molecular epidemiology to better understand the evolution, transmission networks, and host adaptation of *Brucella* in deer.

## 5. Conclusions

Overall, this systematic review and meta‐analysis indicates that the burden of brucellosis in deer in China remains non‐negligible, with a pooled seroprevalence of 16.3% (95% CI: 10.9–22.4) and substantial between‐study variability. Subgroup analyses generally suggested an increasing trend after 2000 and higher estimates in the major deer‐farming areas of northern China; host factors such as deer species, age, and sex, as well as environmental factors such as season, may also be associated with differences in prevalence. Because the number of available stratified studies is limited, further well‐designed studies with complete stratification information are needed to validate these findings and to obtain more stable and comparable estimates. Based on the current evidence, we recommend strengthening deer brucellosis surveillance, further standardizing diagnostic and reporting procedures, and implementing targeted control measures tailored to regional risk levels to reduce infection in deer herds, minimize economic losses, and lower the potential risk of zoonotic transmission.

## Author Contributions

Rui Du and Fei Liu conceived and designed the study and secured funding. Wen‐Tao Xiang drafted and edited the manuscript. Wei Zheng and Jian‐Ming Li contributed to the critical revision of the manuscript. Jia‐Yu Yu and Qiu‐Chi Jiang were responsible for data collection and extraction. Ting Li and Yi‐Fan Zhang established the database. Rui Liang performed the data analysis. All authors have contributed to the revision of the manuscript.

## Funding

This study was funded by the Jilin Provincial Key Research and Development (Grant 20230204010YY) and the National Natural Science Foundation of China (Grant U23A20237).

## Disclosure

All authors have approved the final version of this manuscript.

## Conflicts of Interest

The authors declare no conflicts of interest.

## Supporting Information

Additional supporting information can be found online in the Supporting Information section.

## Supporting information


**Supporting Information** Table S1. PRISMA checklist item. Table S2. The search formulas in PubMed. Table S3. Included studies and quality scores. Table S4. The code in R for this meta‐analysis. Table S5. Egger’s test for publication bias. Table S6. Summary of interaction meta‐regression analyses (detection methods × strata). Figures S1–S32. The results of the meta‐analysis and publication bias of each subgroup. Figure S1. Funnel plot with pseudo 95% confidence limit intervals for the examination of publication bias in the study. Figure S2. Forest plot of the study quality subgroup. Figure S3. Funnel plot with pseudo 95% confidence limit intervals for the examination of publication bias in the sampling years subgroup. Figure S4. Forest plot of the sampling year subgroup. Figure S5. Funnel plot with pseudo 95% confidence limit intervals for the examination of publication bias in the province subgroup. Figure S6. Forest plot of the province subgroup. Figure S7. Funnel plot with pseudo 95% confidence limit intervals for the examination of publication bias in the region subgroup. Figure S8. Forest plot of the region subgroup. Figure S9. Funnel plot with pseudo 95% confidence limit intervals for the examination of publication bias in the detection method subgroup. Figure S10. Forest plot of the detection method subgroup. Figure S11. Funnel plot with pseudo 95% confidence limit intervals for the examination of publication bias in the variety subgroup. Figure S12. Forest plot of the variety subgroup. Figure S13. Funnel plot with pseudo 95% confidence limit intervals for the examination of publication bias in the age subgroup. Figure S14. Forest plot of the age subgroup. Figure S15. Funnel plot with pseudo 95% confidence limit intervals for the examination of publication bias in the gender subgroup. Figure S16. Forest plot of the gender subgroup. Figure S17. Funnel plot with pseudo 95% confidence limit intervals for the examination of publication bias in the season subgroup. Figure S18. Forest plot of the season subgroup. Figure S19. Funnel plot with pseudo 95% confidence limit intervals for the examination of publication bias in the longitude subgroup. Figure S20. Forest plot of the longitude subgroup. Figure S21. Funnel plot with pseudo 95% confidence limit intervals for the examination of publication bias in the latitude subgroup. Figure S22. Forest plot of the latitude subgroup. Figure S23. Funnel plot with pseudo 95% confidence limit intervals for the examination of publication bias in the altitude subgroup. Figure S24. Forest plot of the altitude subgroup. Figure S25. Funnel plot with pseudo 95% confidence limit intervals for the examination of publication bias in the rainfall subgroup. Figure S26. Forest plot of the rainfall subgroup. Figure S27. Funnel plot with pseudo 95% confidence limit intervals for the examination of publication bias in the humidity subgroup. Figure S28. Forest plot of the humidity subgroup. Figure S29. Funnel plot with pseudo 95% confidence limit intervals for the examination of publication bias in the average annual temperature subgroup. Figure S30. Forest plot of the average annual temperature subgroup. Figure S31. Funnel plot with pseudo 95% confidence limit intervals for the examination of publication bias in the climate subgroup. Figure S32. Forest plot of the climate subgroup.

## Data Availability

The datasets used in this work are accessible upon reasonable request from the corresponding author.
