# Supplementary material for: Epidemiology of Brucellosis Among Deer in China From 1978 to 2025: A Systematic Review and Meta‐Analysis
Source: Transbound Emerg Dis. 2026 May 20;2026:9727215. doi: 10.1155/tbed/9727215 (PMC13189476; doi:10.1155/tbed/9727215)
Supplement: Supplementary file 1 — Supporting Information Table S1. PRISMA checklist item. Table S2. The search formulas in PubMed. Table S3. Included studies and quality scores. Table S4. The code in R for this meta‐analysis. Table S5. Egger’s test for publication bias. Table S6. Summary of interaction meta‐regression analyses (detection methods × strata). Figures S1–S32. The results of the meta‐analysis and publication bias of each subgroup. Figure S1. Funnel plot with pseudo 95% confidence limit intervals for the examination of publication bias in the study. Figure S2. Forest plot of the study quality subgroup. Figure S3. Funnel plot with pseudo 95% confidence limit intervals for the examination of publication bias in the sampling years subgroup. Figure S4. Forest plot of the sampling year subgroup. Figure S5. Funnel plot with pseudo 95% confidence limit intervals for the examination of publication bias in the province subgroup. Figure S6. Forest plot of the province subgroup. Figure S7. Funnel plot with pseudo 95% confidence limit intervals for the examination of publication bias in the region subgroup. Figure S8. Forest plot of the region subgroup. Figure S9. Funnel plot with pseudo 95% confidence limit intervals for the examination of publication bias in the detection method subgroup. Figure S10. Forest plot of the detection method subgroup. Figure S11. Funnel plot with pseudo 95% confidence limit intervals for the examination of publication bias in the variety subgroup. Figure S12. Forest plot of the variety subgroup. Figure S13. Funnel plot with pseudo 95% confidence limit intervals for the examination of publication bias in the age subgroup. Figure S14. Forest plot of the age subgroup. Figure S15. Funnel plot with pseudo 95% confidence limit intervals for the examination of publication bias in the gender subgroup. Figure S16. Forest plot of the gender subgroup. Figure S17. Funnel plot with pseudo 95% confidence limit intervals for the examination of publication bias in the season subgroup. F [file TBED-2026-9727215-s001.docx]

**TABLE S1. PRISMA Checklist item.**

| **Section/topic** | **#** | **Checklist item** | **Reported on page #** |
| --- | --- | --- | --- |
| **TITLE** |  |  |  |
| Title | 1 | Epidemiology of Brucellosis among Deer in China from 1978 to 2025—A Systematic Review and Meta-Analysis | 1 |
| **ABSTRACT** |  |  |  |
| Structured summary | 2 | Provide a structured summary including, as applicable: background; objectives; data sources; study eligibility criteria, participants, and interventions; study appraisal and synthesis methods; results; limitations; conclusions and implications of key findings; systematic review registration number. | 1-2 |
| **INTRODUCTION** |  |  |  |
| Rationale | 3 | Describe the rationale for the review in the context of what is already known. | 2-4 |
| Objectives | 4 | Provide an explicit statement of questions being addressed with reference to participants, interventions, comparisons, outcomes, and study design (PICOS). | 4 |
| **METHODS** |  |  |  |
| Protocol and registration | 5 | Indicate if a review protocol exists, if and where it can be accessed (e.g., Web address), and, if available, provide registration information including registration number. | 5 |
| Eligibility criteria | 6 | Specify study characteristics (e.g., PICOS, length of follow-up) and report characteristics (e.g., years considered, language, publication status) used as criteria for eligibility, giving rationale. | 5 |
| Information sources | 7 | Describe all information sources (e.g., databases with dates of coverage, contact with study authors to identify additional studies) in the search and date last searched. | 5 |
| Search | 8 | Present full electronic search strategy for at least one database, including any limits used, such that it could be repeated. | 5 |
| Study selection | 9 | State the process for selecting studies (i.e., screening, eligibility, included in systematic review, and, if applicable, included in the meta-analysis). | 5 |
| Data collection process | 10 | Describe method of data extraction from reports (e.g., piloted forms, independently, in duplicate) and any processes for obtaining and confirming data from investigators. | 5 |
| Data items | 11 | List and define all variables for which data were sought (e.g., PICOS, funding sources) and any assumptions and simplifications made. | 6 |
| Risk of bias in individual studies | 12 | Describe methods used for assessing risk of bias of individual studies (including specification of whether this was done at the study or outcome level), and how this information is to be used in any data synthesis. | 6 |
| Summary measures | 13 | State the principal summary measures (e.g., risk ratio, difference in means). | 6-7 |
| Synthesis of results | 14 | Describe the methods of handling data and combining results of studies, if done, including measures of consistency (e.g., I^2^) for each meta-analysis. | 6-7 |
| Risk of bias across studies | 15 | Specify any assessment of risk of bias that may affect the cumulative evidence (e.g., publication bias, selective reporting within studies). | 6-7 |
| Additional analyses | 16 | Describe methods of additional analyses (e.g., sensitivity or subgroup analyses, meta-regression), if done, indicating which were pre-specified. | 6-7 |
| **RESULTS** |  |  |  |
| Study selection | 17 | Give numbers of studies screened, assessed for eligibility, and included in the review, with reasons for exclusions at each stage, ideally with a flow diagram. | 7-8; FIGURE 1 |
| Study characteristics | 18 | For each study, present characteristics for which data were extracted (e.g., study size, PICOS, follow-up period) and provide the citations. | TABLE 2-4 |
| Risk of bias within studies | 19 | Present data on risk of bias of each study and, if available, any outcome level assessment (see item 12). | 7-8; TABLE 2; Supplementary TABLE S3 |
| Results of individual studies | 20 | For all outcomes considered (benefits or harms), present, for each study: (a) simple summary data for each intervention group (b) effect estimates and confidence intervals, ideally with a forest plot. | FIGURE 2; TABLE 3-5; Supplementary Figures S1-S32 |
| Synthesis of results | 21 | Present results of each meta-analysis done, including confidence intervals and measures of consistency. | 8-12; FIGURE 2-3 |
| Risk of bias across studies | 22 | Present results of any assessment of risk of bias across studies (see Item 15). | 8; FIGURE 3-5; Supplementary TABLE S5 |
| Additional analysis | 23 | Give results of additional analyses, if done (e.g., sensitivity or subgroup analyses, meta-regression [see Item 16]). | 8-12; TABLE 5; Supplementary TABLE S6; Supplementary FIGURE S1-S32 |
| **DISCUSSION** |  |  |  |
| Summary of evidence | 24 | Summarize the main findings including the strength of evidence for each main outcome; consider their relevance to key groups (e.g., healthcare providers, users, and policy makers). | 12-23 |
| Limitations | 25 | Discuss limitations at study and outcome level (e.g., risk of bias), and at review-level (e.g., incomplete retrieval of identified research, reporting bias). | 24 |
| Conclusions | 26 | Provide a general interpretation of the results in the context of other evidence, and implications for future research. | 24 |
| **FUNDING** |  |  |  |
| Funding | 27 | Describe sources of funding for the systematic review and other support (e.g., supply of data); role of founders for the systematic review. | 25 |

*From:* Moher D, Liberati A, Tetzlaff J, Altman DG, The PRISMA Group (2009). Preferred Reporting Items for Systematic Reviews and Meta-Analyses: The PRISMA Statement. PLoS Med 6(6): e1000097. doi:10.1371/journal.pmed1000097

For more information, visit: **www.prisma-statement.org**.

**TABLE S2. The search formulas in PubMed.**

| **PubMed** | **((("Deer"[Mesh]) OR (Deers)) AND ((((((((((((((((((("Brucellosis"[Mesh]) OR (Brucelloses)) OR (*Brucella* Infection)) OR (*Brucella* Infections)) OR (Infection, *Brucella*)) OR (Malta Fever)) OR (Fever, Malta)) OR (Undulant Fever)) OR (Fever, Undulant)) OR (Rock Fever)) OR (Fever, Rock)) OR (Cyprus Fever)) OR (Fever, Cyprus)) OR (Gibraltar Fever)) OR (Fever, Gibraltar)) OR (Brucellosis, Pulmonary)) OR (Brucelloses, Pulmonary)) OR (Pulmonary Brucelloses)) OR (Pulmonary Brucellosis))) AND (((((("China"[Mesh]) OR (People's Republic of China)) OR (Inner Mongolia)) OR (Manchuria)) OR (Sinkiang)) OR (Mainland China))** |
| --- | --- |

**TABLE S3. Included studies and quality scores.**

| NO. | Reference ID | No. tested | No. positive | Prevalence | Random sampling or not | The timing of the sampling clear or not | Sampled method detailedly or not | The detection method clear or not | Four or more risk factors or not | Score | Quality  level |
| --- | --- | --- | --- | --- | --- | --- | --- | --- | --- | --- | --- |
| 1 | **Zhou et al. (1978)** | **92** | **20** | **0.217391304** | **N** | **N** | **N** | **Y** | **Y** | **2** | **middle** |
| 2 | **Jia (1980)** | **397** | **294** | **0.740554156** | **N** | **Y** | **N** | **Y** | **Y** | **3** | **middle** |
| 3 | **Zhang and Qi (1982)** | **390** | **13** | **0.033333333** | **N** | **Y** | **N** | **Y** | **Y** | **3** | **middle** |
| 4 | **Jin (1983)** | **245** | **148** | **0.604081633** | **Y** | **Y** | **N** | **Y** | **Y** | **4** | **high** |
| 5 | **Ma (1983)** | **173** | **31** | **0.179190751** | **N** | **Y** | **Y** | **Y** | **Y** | **4** | **high** |
| 6 | **Song et al. (1987)** | **523** | **59** | **0.112810707** | **N** | **Y** | **N** | **Y** | **Y** | **3** | **middle** |
| 7 | **Jin (1991)** | **47** | **4** | **0.085106383** | **Y** | **Y** | **N** | **Y** | **Y** | **4** | **high** |
| 8 | **Li et al. (1991)** | **12748** | **94** | **0.007373706** | **N** | **Y** | **N** | **Y** | **Y** | **3** | **middle** |
| 9 | **Bao et al. (1993)** | **27** | **12** | **0.444444444** | **N** | **Y** | **Y** | **Y** | **Y** | **4** | **high** |
| 10 | **Qu et al. (1996)** | **2840** | **3** | **0.001056338** | **N** | **Y** | **N** | **Y** | **Y** | **3** | **middle** |
| 11 | **Chen et al. (1997)** | **300** | **42** | **0.14** | **N** | **Y** | **N** | **Y** | **Y** | **3** | **middle** |
| 12 | **Li et al. (1997)** | **2139** | **0** | **0** | **N** | **Y** | **N** | **Y** | **Y** | **3** | **middle** |
| 13 | **Jiang et al. (2000)** | **504** | **24** | **0.047619048** | **N** | **Y** | **N** | **N** | **Y** | **2** | **middle** |
| 14 | **Zhang et al. (2002)** | **728** | **20** | **0.027472527** | **N** | **Y** | **Y** | **Y** | **Y** | **4** | **high** |
| 15 | **Zhao et al. (2002)** | **580** | **304** | **0.524137931** | **N** | **Y** | **N** | **Y** | **Y** | **3** | **middle** |
| 16 | **Chao (2002)** | **206** | **1** | **0.004854369** | **N** | **Y** | **N** | **Y** | **Y** | **3** | **middle** |
| 17 | **Mokhtar et al. (2002)** | **531** | **193** | **0.36346516** | **N** | **Y** | **N** | **Y** | **Y** | **3** | **middle** |
| 18 | **Cui et al. (2004)** | **9** | **7** | **0.777777778** | **N** | **Y** | **N** | **Y** | **Y** | **3** | **middle** |
| 19 | **Zeng et al. (2004)** | **173** | **7** | **0.040462428** | **N** | **Y** | **Y** | **Y** | **Y** | **4** | **high** |
| 20 | **Huang et al. (2004)** | **3630** | **723** | **0.199173554** | **Y** | **Y** | **N** | **Y** | **Y** | **4** | **high** |
| 21 | **Li et al. (2005)** | **874** | **112** | **0.128146453** | **Y** | **N** | **N** | **Y** | **Y** | **3** | **middle** |
| 22 | **Zhang et al. (2006)** | **6779** | **1968** | **0.290308305** | **N** | **Y** | **N** | **Y** | **Y** | **3** | **middle** |
| 23 | **Meng et al. (2006)** | **300** | **47** | **0.156666667** | **N** | **N** | **N** | **Y** | **Y** | **2** | **middle** |
| 24 | **Zhou and Fan (2007)** | **3100** | **206** | **0.066451613** | **N** | **Y** | **N** | **Y** | **Y** | **3** | **middle** |
| 25 | **Yan et al. (2007)** | **3644** | **210** | **0.057628979** | **Y** | **Y** | **Y** | **Y** | **Y** | **5** | **high** |
| 26 | **Li et al. (2007)** | **1014** | **143** | **0.141025641** | **Y** | **Y** | **N** | **Y** | **Y** | **4** | **high** |
| 27 | **Dolkun et al. (2008)** | **27** | **20** | **0.76** | **N** | **Y** | **Y** | **Y** | **Y** | **4** | **high** |
| 28 | **Yao and Yu (2009)** | **541** | **144** | **0.266173752** | **N** | **N** | **N** | **Y** | **Y** | **2** | **middle** |
| 29 | **Zhao and Zhang (2010)** | **85** | **20** | **0.235294118** | **N** | **N** | **N** | **Y** | **Y** | **2** | **middle** |
| 30 | **Yu et al. (2011)** | **1055** | **213** | **0.201895735** | **N** | **N** | **N** | **Y** | **Y** | **2** | **middle** |
| 31 | **Wang (2011)** | **1872** | **101** | **0.053952991** | **N** | **Y** | **N** | **Y** | **Y** | **3** | **middle** |
| 32 | **Mijiti and Dolkun (2012)** | **61** | **48** | **0.786885246** | **N** | **N** | **Y** | **Y** | **Y** | **3** | **middle** |
| 33 | **Hasibat et al. (2012)** | **72** | **11** | **0.152777778** | **N** | **Y** | **N** | **Y** | **Y** | **3** | **middle** |
| 34 | **Wu et al. (2015)** | **420** | **66** | **0.157142857** | **N** | **N** | **N** | **Y** | **Y** | **2** | **middle** |
| 35 | **Wu (2015)** | **254** | **26** | **0.102362205** | **N** | **N** | **N** | **Y** | **Y** | **2** | **middle** |
| 36 | **Liu et al. (2017)** | **263** | **99** | **0.376425856** | **N** | **Y** | **N** | **Y** | **Y** | **3** | **middle** |
| 37 | **Liu et al. (2017)** | **458** | **59** | **0.128820961** | **Y** | **Y** | **N** | **Y** | **Y** | **4** | **high** |
| 38 | **Shi et al. (2019)** | **674** | **163** | **0.241839763** | **N** | **N** | **N** | **Y** | **Y** | **2** | **middle** |
| 39 | **Wang and Yan (2020)** | **98** | **25** | **0.255102041** | **N** | **Y** | **N** | **Y** | **Y** | **3** | **middle** |
| 40 | **Wu et al. (2020)** | **72** | **2** | **0.027777778** | **Y** | **Y** | **Y** | **Y** | **Y** | **5** | **high** |
| 41 | **Li et al. (2021)** | **10** | **2** | **0.2** | **N** | **Y** | **N** | **Y** | **Y** | **3** | **middle** |
| 42 | **Zhong et al. (2022)** | **150** | **0** | **0** | **N** | **N** | **N** | **Y** | **Y** | **2** | **middle** |
| 43 | **Yuan et al. (2023)** | **56** | **15** | **0.267857143** | **N** | **Y** | **N** | **Y** | **Y** | **3** | **middle** |
| 44 | **Li (2023)** | **783** | **41** | **0.052362708** | **Y** | **Y** | **Y** | **Y** | **Y** | **5** | **high** |
| 45 | **Wang (2023)** | **1240** | **72** | **0.058064516** | **Y** | **N** | **N** | **Y** | **Y** | **3** | **middle** |
| 46 | **Liao and Liu (2024)** | **45** | **0** | **0** | **Y** | **Y** | **N** | **Y** | **Y** | **4** | **high** |
| 47 | **Tian et al. (2025)** | **4470** | **354** | **0.079194631** | **Y** | **N** | **Y** | **Y** | **Y** | **4** | **high** |

**Y*: Yes; N*: No.**

**Quality level*：High (4-5); Middle (2-3); Low (0-1).**

**TABLE S4. The code in R for this meta-analysis.**

| No transformation (PRAW) | rate<-transform(m1, r= event/n)  shapiro.test(rate$r) |
| --- | --- |
| Logarithmic conversion (PLN) | **rate<-transform(m1, log=log(event/n))**  **shapiro.test(rate$log)** |
| Logit transformation (PLOGIT) | **rate<-transform(m1, logit=log((event/n)/(1-event/n)))**  **shapiro.test(rate$logit)** |
| Arcsine transformation (PAS) | **rate<-transform(m1, arcsin.size=asin(sqrt(event/(n+1))))**  **shapiro.test(rate$arcsin)** |
| Double-arcsine transformation (PFT) | **rate<-transform(m1,darcsin=0.5*(asin(sqrt(event/(n+1)))+asin((sqrt(event+1)/(n+1)))))**  **shapiro.test(rate$darcsin)** |
| Forest plots | **forest (meta1, xlim=c(-0.2, 1) )** |
| Funnel chart | **funnel(meta1)** |
| Egger's test | **metabias(meta1, method="linreg")** |
| The sensitivity analysis | **metainf(meta1, pooled = "random") forest(meta_info, xlim = c(0.15, 0.45))** |
| Subgroup analysis | **meta1<-metaprop(event, n, study, data=rate, sm="PFT", incr=0.5, allincr=TRUE, addincr=FALSE, title="", byvar= subgroup title, print.byvar=TRUE)** |
| Meta-regression analysis | **metareg (meta1, ~covariate title)** |

**TABLE S5. Egger’s test for publication bias.**

| bias | se. bias | t | df | *p*-value |
| --- | --- | --- | --- | --- |
| 6.9803 | **3.0746** | **2.27** | **45** | **0.0280** |

**TABLE S6. Summary of interaction meta-regression analyses (Detection methods × strata).**

| Interaction model | k | QM (df) | *p*-value | R² (%) | Residual I² (%) |
| --- | --- | --- | --- | --- | --- |
| Detection methods × Region | **47** | **17.9841 (17)** | **0.3898** | **0.00** | **99.41** |
| Detection methods × Variety | **47** | **10.8825(13)** | **0.6207** | **0.00** | **99.26** |
| Detection methods × Study period | **32** | **8.6950 (12)** | **0.7287** | **0.00** | **99.55** |
| Detection methods × Season | **20** | **10.0717 (10)** | **0.4342** | **0.00** | **99.20** |
| Detection methods × Age | **13** | **2.4568 (7)** | **0.9303** | **0.00** | **98.95** |
| Detection methods × Gender | **22** | **3.6881 (7)** | **0.8149** | **0.00** | **99.39** |
| Detection methods × Quality points | **45** | **7.6651 (11)** | **0.7430** | **0.00** | **99.53** |

**QM denotes the omnibus test of moderators from mixed-effects meta-regression. R² represents the proportion of between-study heterogeneity explained (pseudo-R²).**

**FIGURE S1. Funnel plot with pseudo 95% confidence limit intervals for the examination of publication bias in the study quality subgroup.**


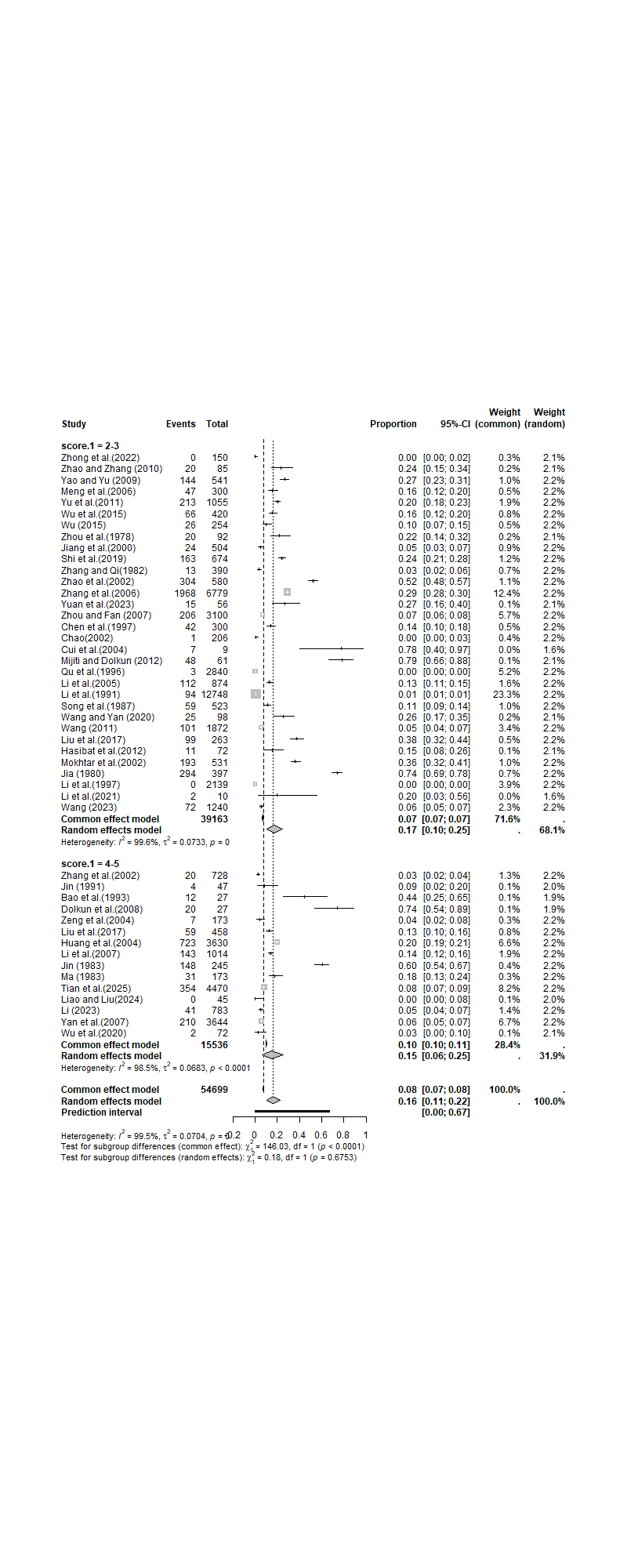


**FIGURE S2. Forest plot of the study quality subgroup.**

**FIGURE S3. Funnel plot with pseudo 95% confidence limit intervals for the examination of publication bias in the sampling years subgroup.**


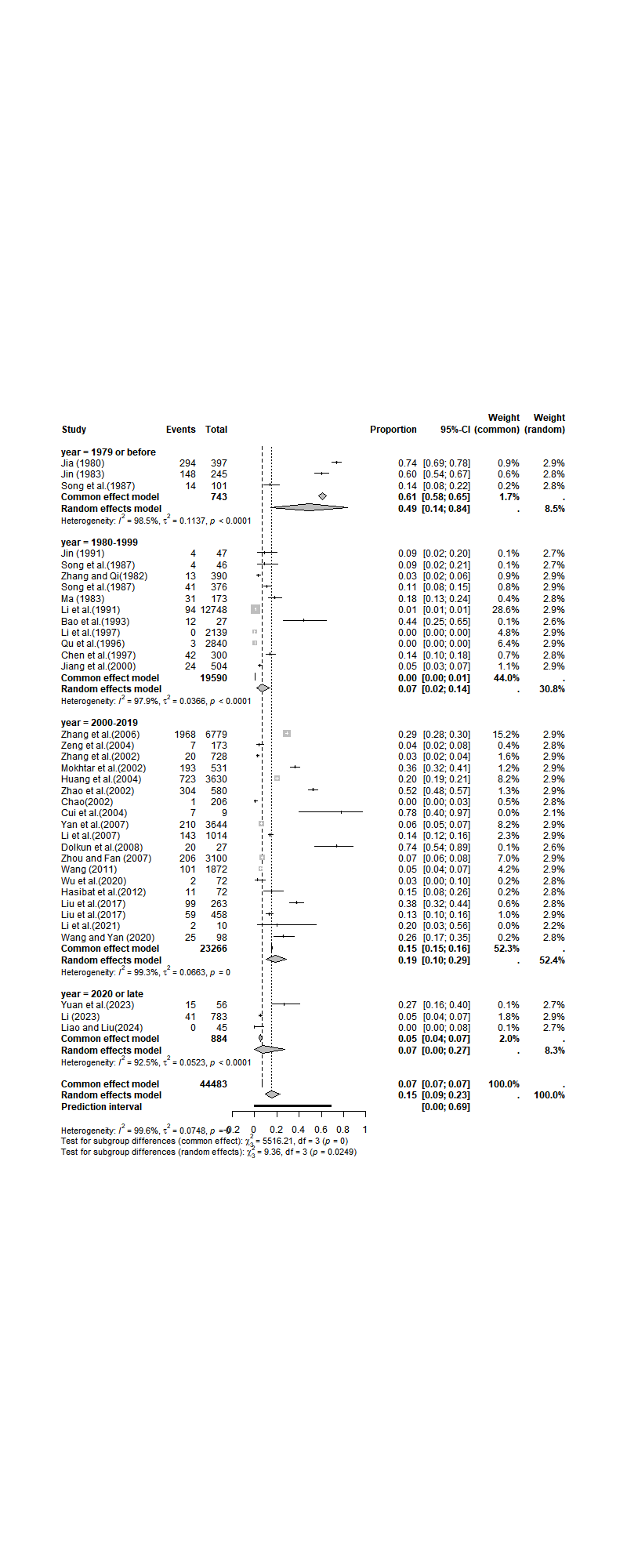


**FIGURE S4. Forest plot of the sampling year subgroup.**

**FIGURE S5. Funnel plot with pseudo 95% confidence limit intervals for the examination of publication bias in the province subgroup.**


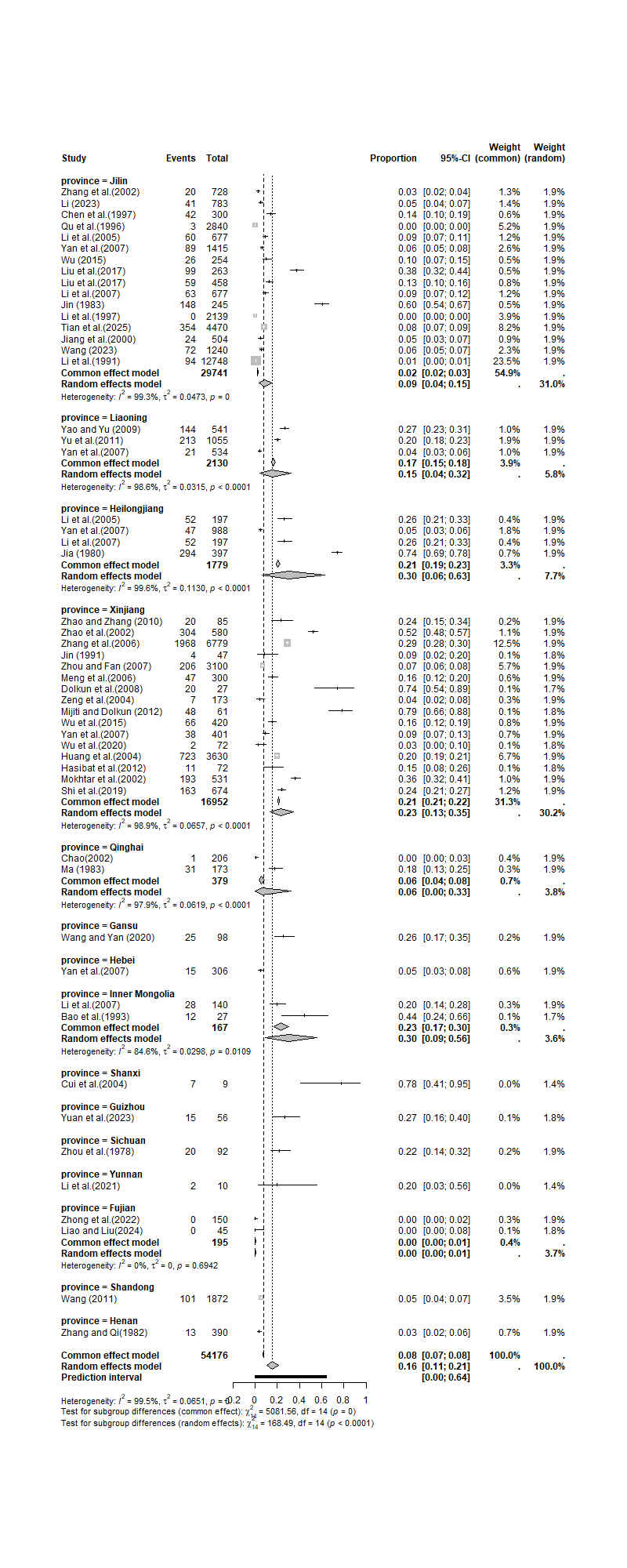


**FIGURE S6. Forest plot of the province subgroup.**

**FIGURE S7. Funnel plot with pseudo 95% confidence limit intervals for the examination of publication bias in the region subgroup.**


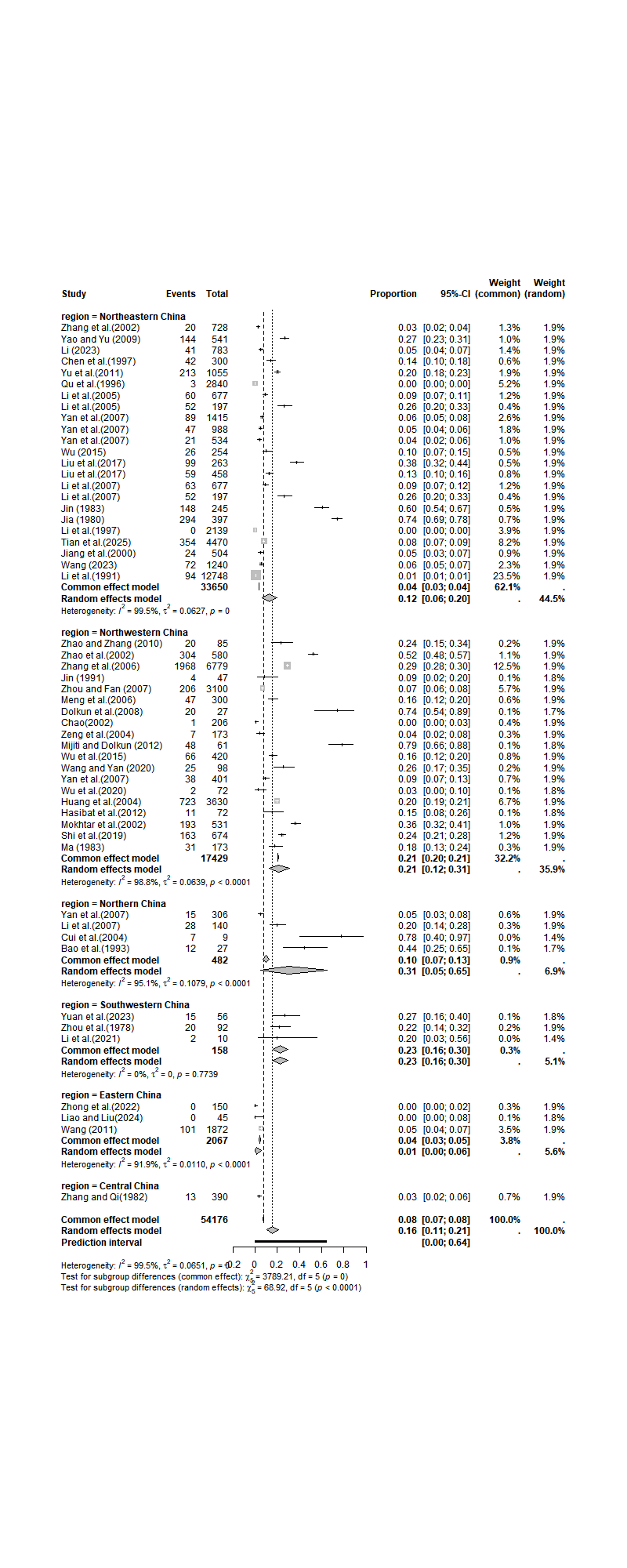


**FIGURE S8. Forest plot of the region subgroup.**

**FIGURE S9. Funnel plot with pseudo 95% confidence limit intervals for the examination of publication bias in the detection method subgroup.**


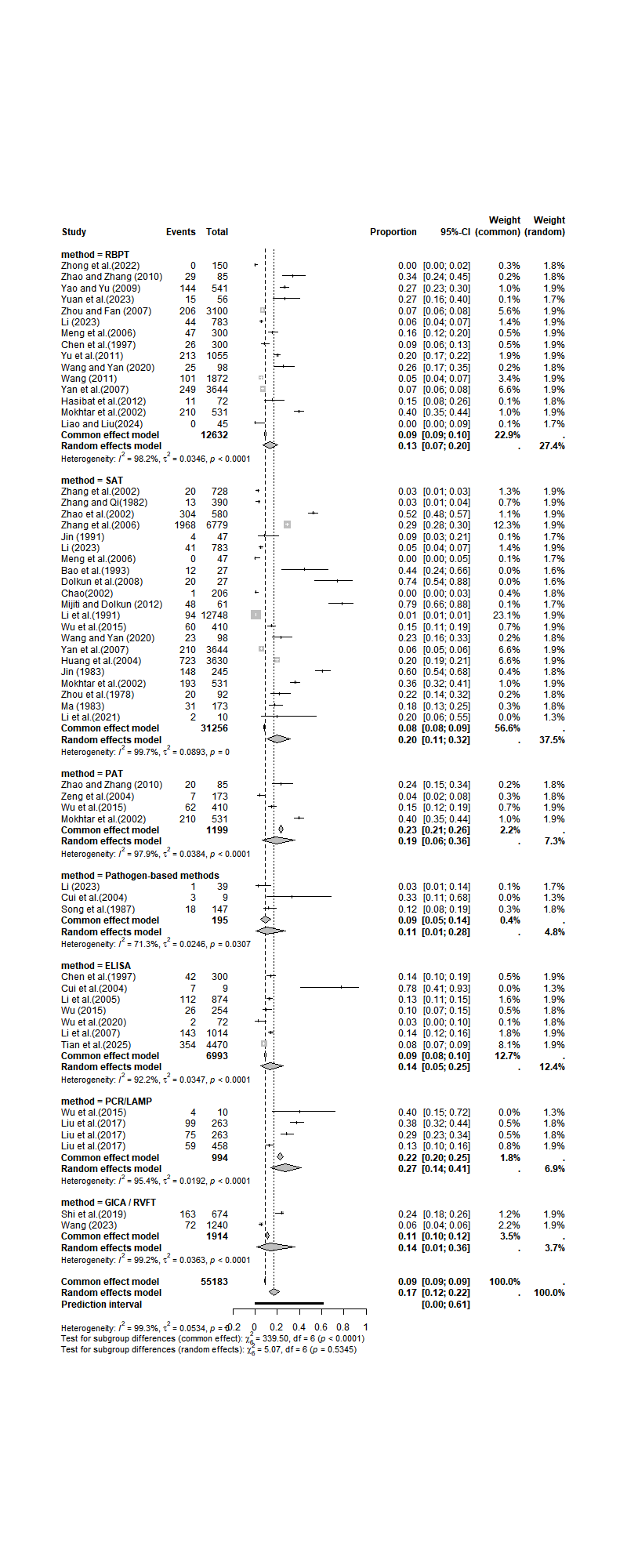


**FIGURE S10. Forest plot of the detection method subgroup.**

**FIGURE S11. Funnel plot with pseudo 95% confidence limit intervals for the examination of publication bias in the variety subgroup.**


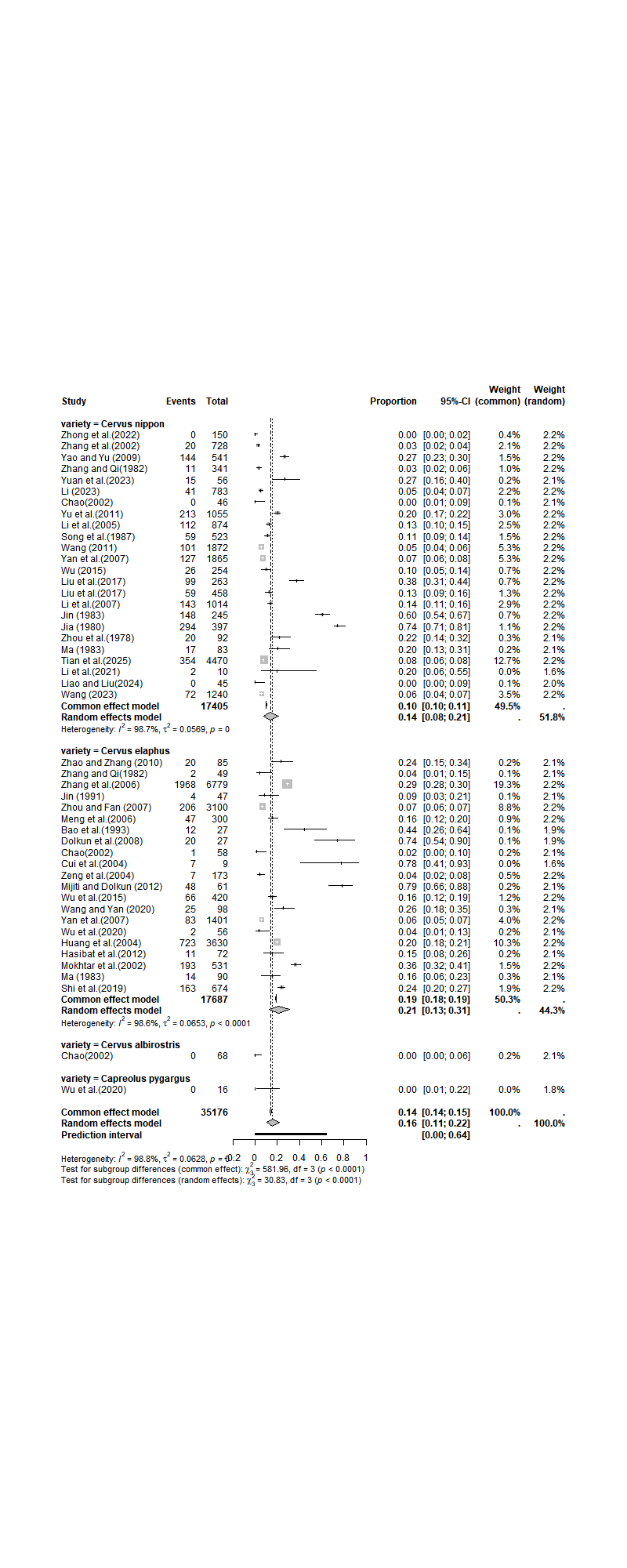


**FIGURE S12. Forest plot of the variety subgroup.**

**FIGURE S13. Funnel plot with pseudo 95% confidence limit intervals for the examination of publication bias in the age subgroup.**


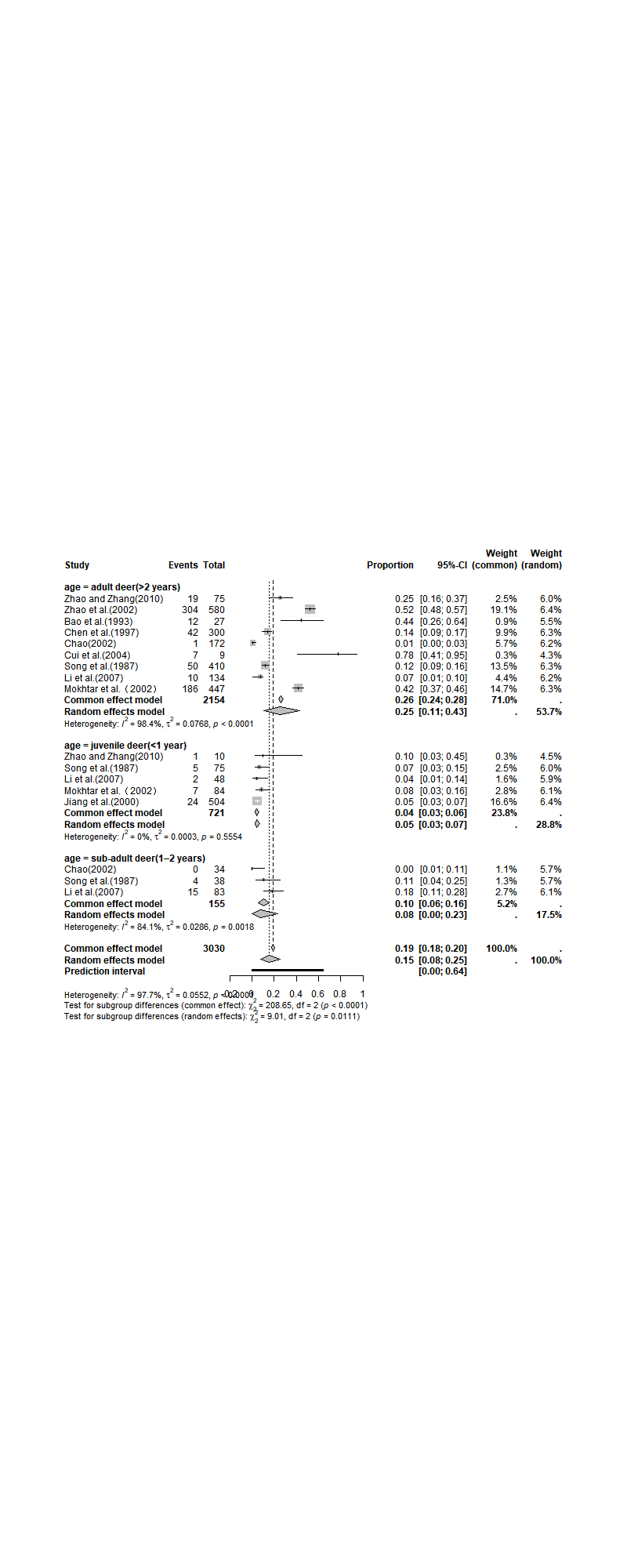


**FIGURE S14. Forest plot of the age subgroup.**

**FIGURE S15. Funnel plot with pseudo 95% confidence limit intervals for the examination of publication bias in the gender subgroup.**


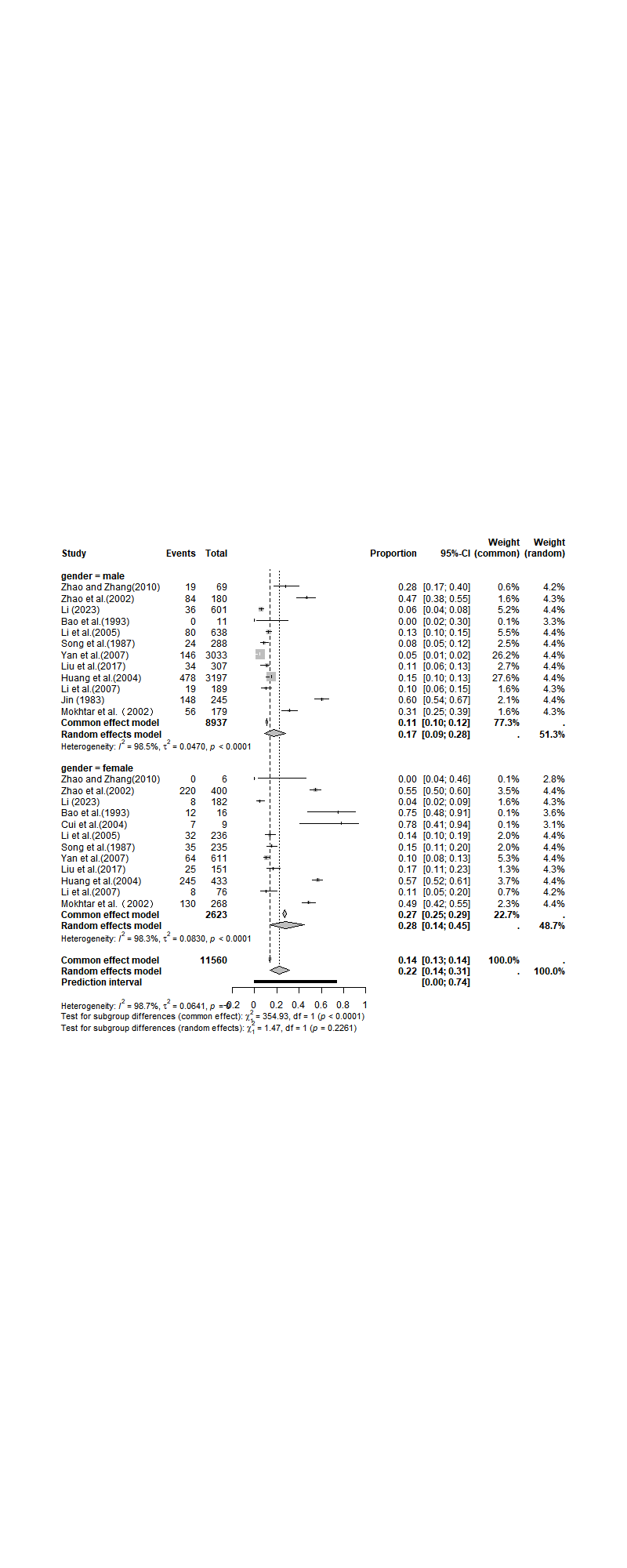


**FIGURE S16. Forest plot of the gender subgroup.**

**FIGURE S17. Funnel plot with pseudo 95% confidence limit intervals for the examination of publication bias in the season subgroup.**


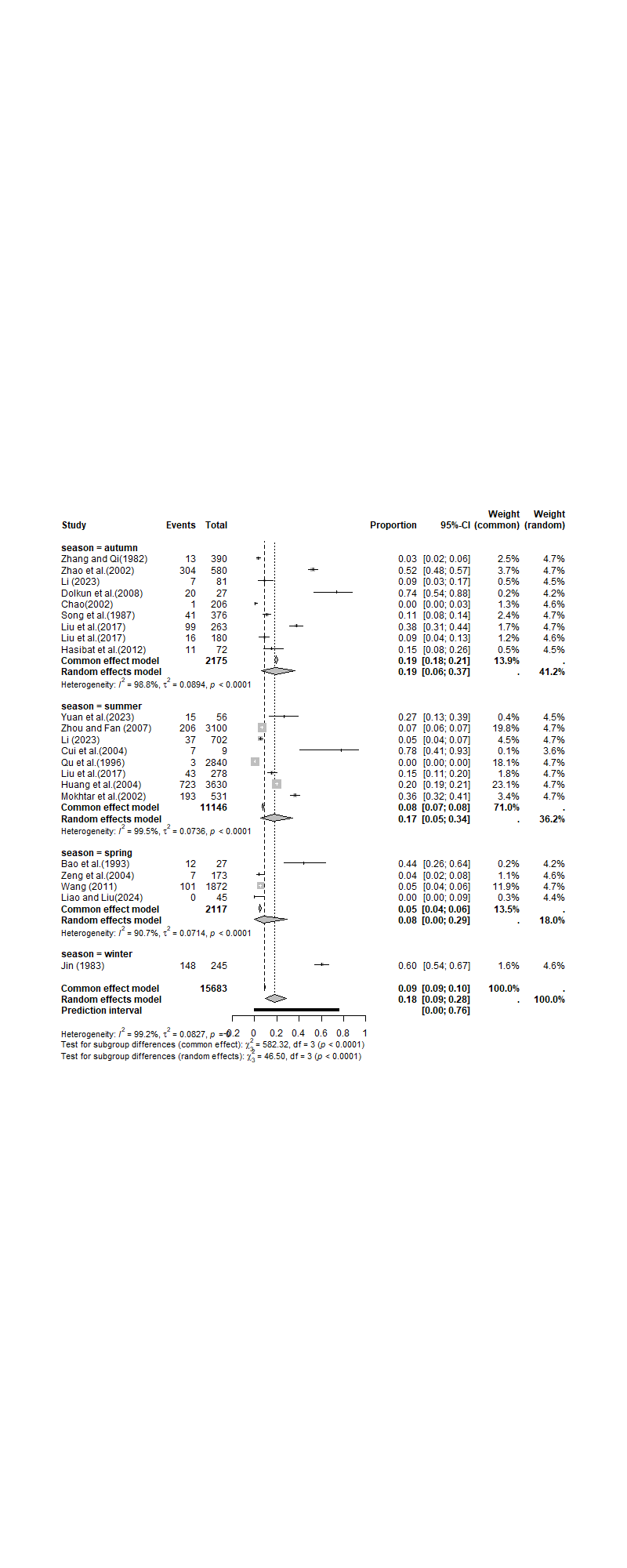


**FIGURE S18. Forest plot of the season subgroup.**

**FIGURE S19. Funnel plot with pseudo 95% confidence limit intervals for the examination of publication bias in the longitude subgroup.**


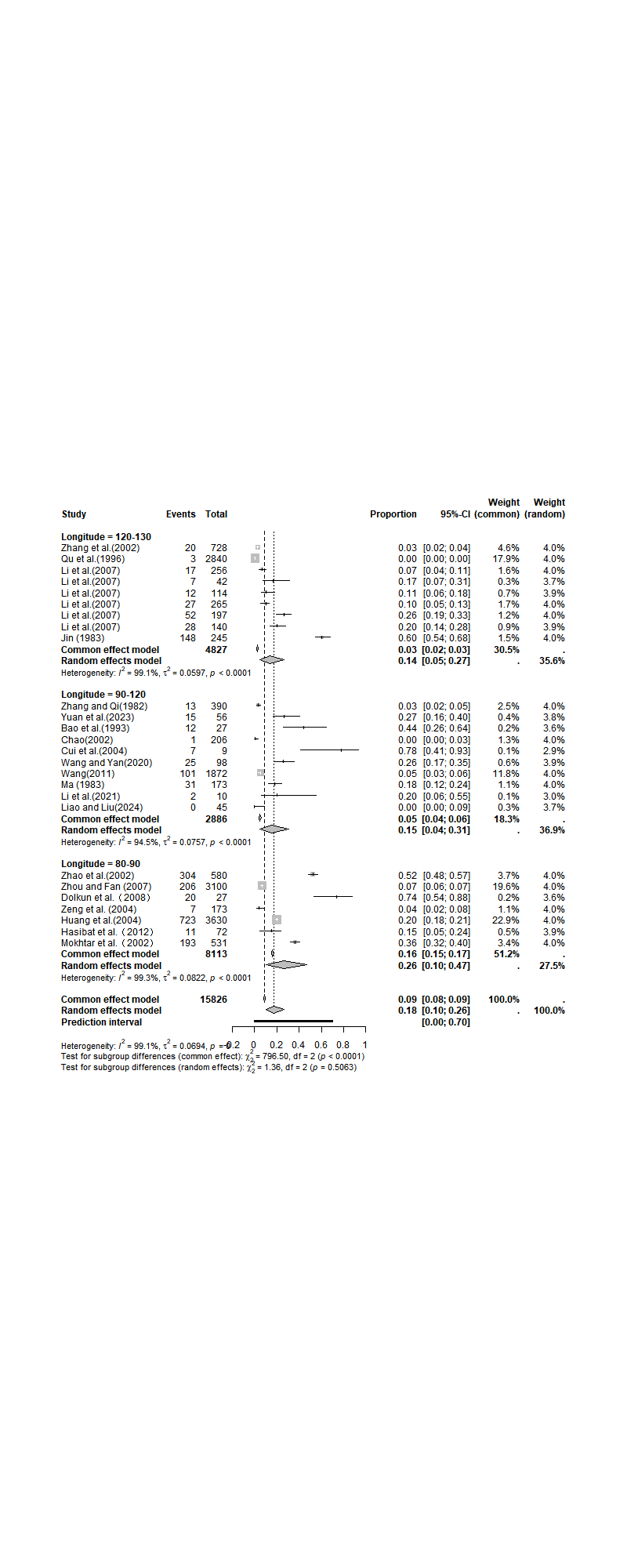


**FIGURE S20. Forest plot of the longitude subgroup.**

**FIGURE S21. Funnel plot with pseudo 95% confidence limit intervals for the examination of publication bias in the latitude subgroup.**


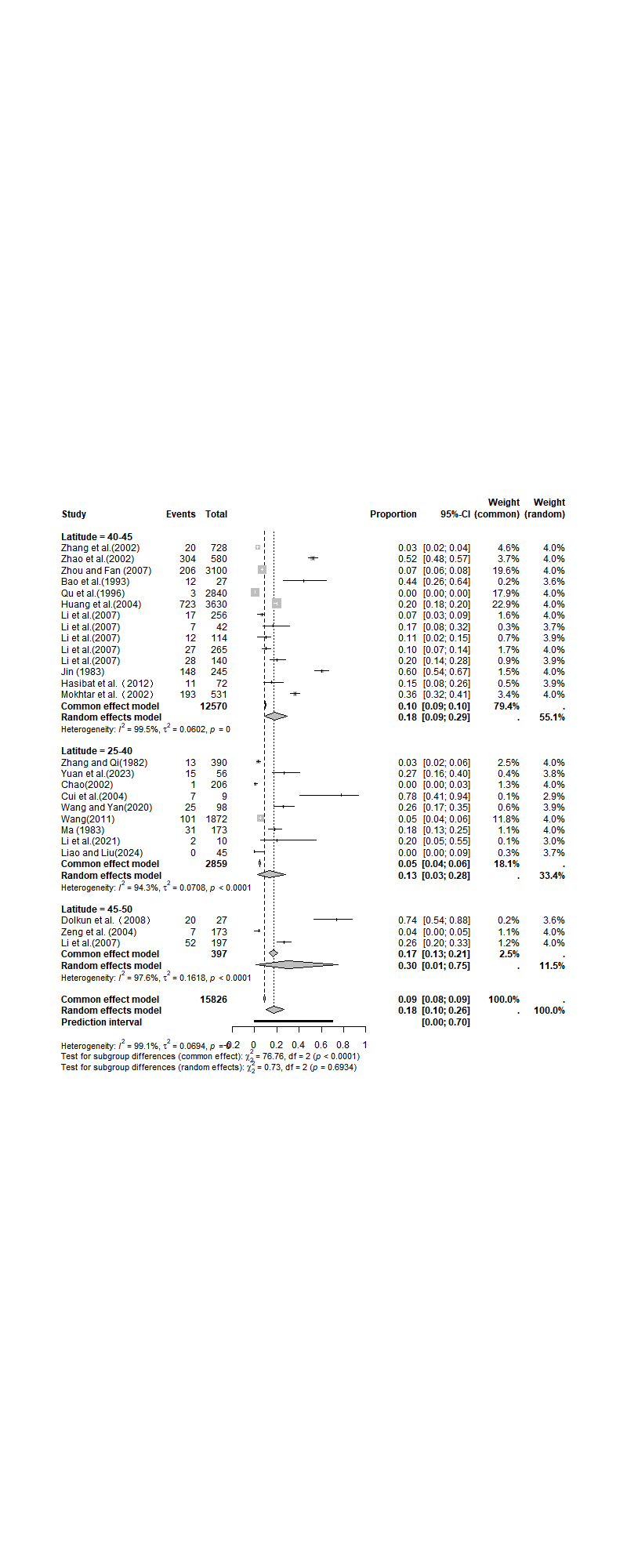


**FIGURE S22. Forest plot of the latitude subgroup.**

**FIGURE S23. Funnel plot with pseudo 95% confidence limit intervals for the examination of publication bias in the altitude subgroup.**


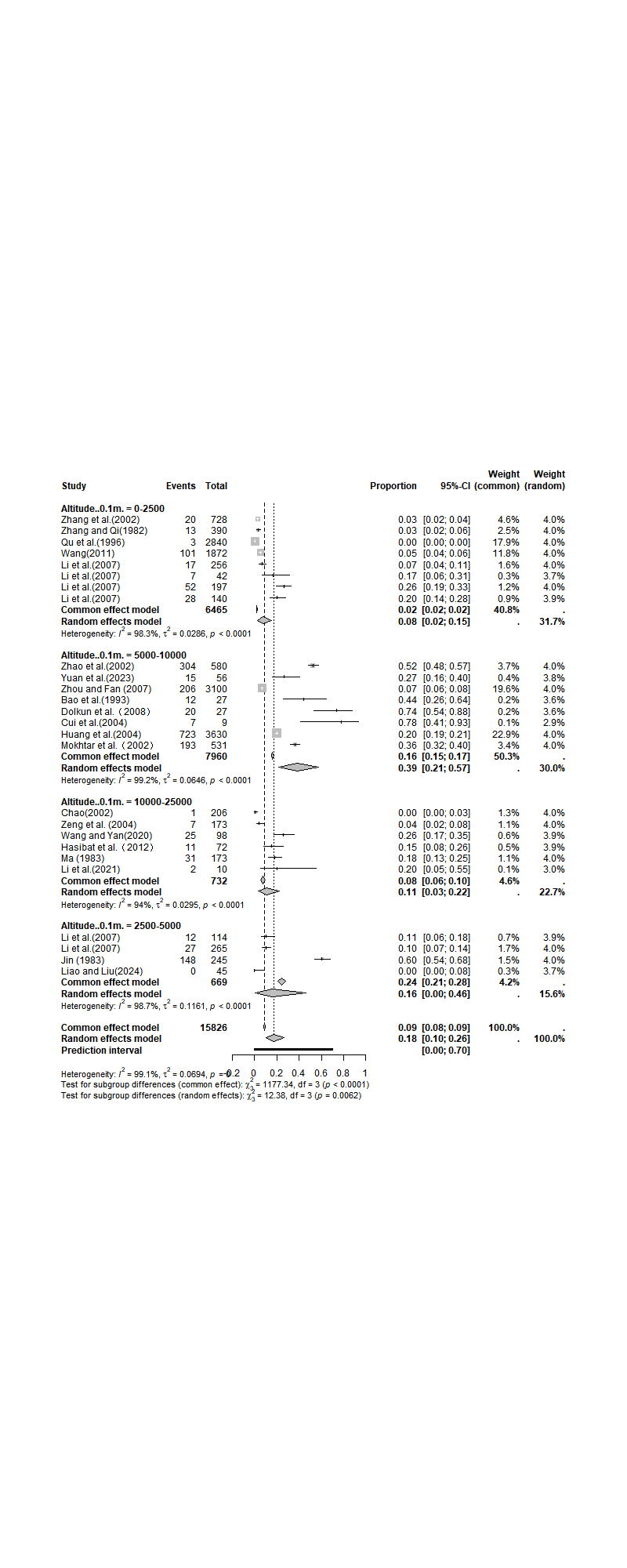


**FIGURE S24. Forest plot of the altitude subgroup.**

**FIGURE S25. Funnel plot with pseudo 95% confidence limit intervals for the examination of publication bias in the rainfall subgroup.**


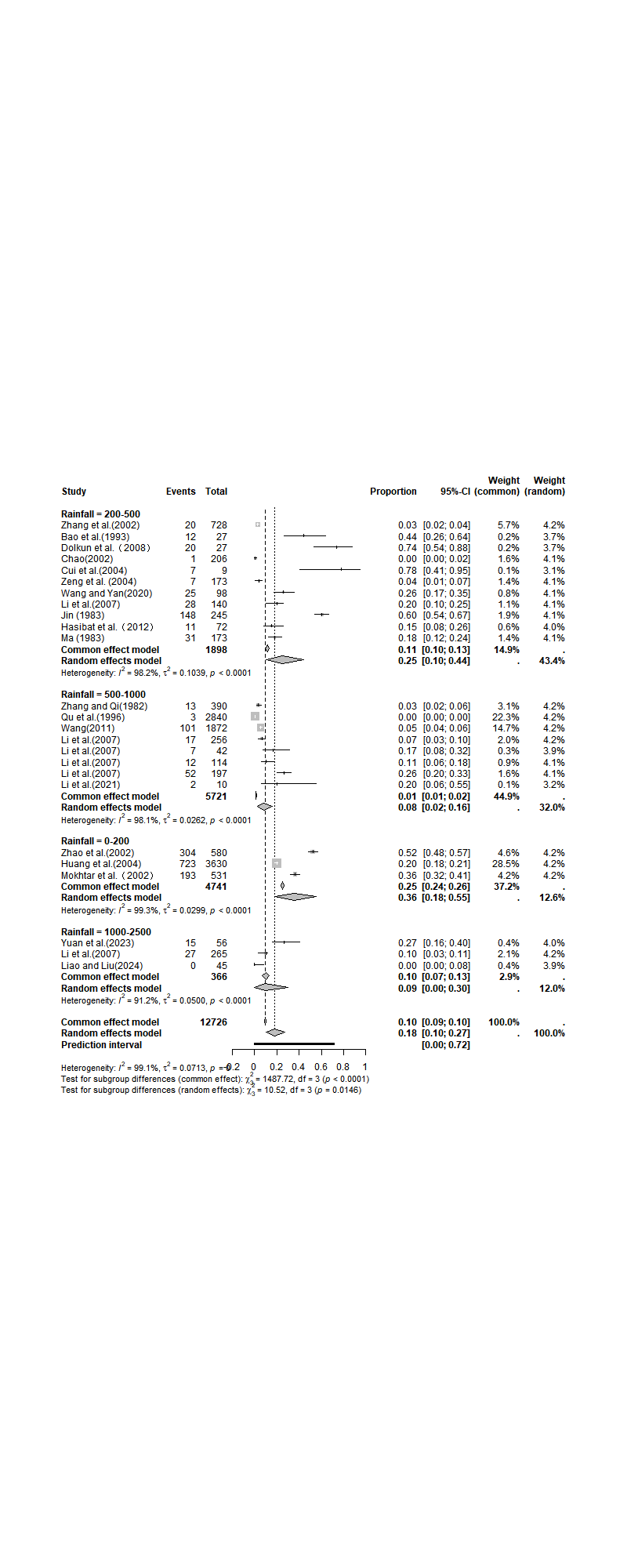


**FIGURE S26. Forest plot of the rainfall subgroup.**

**FIGURE S27. Funnel plot with pseudo 95% confidence limit intervals for the examination of publication bias in the humidity subgroup.**


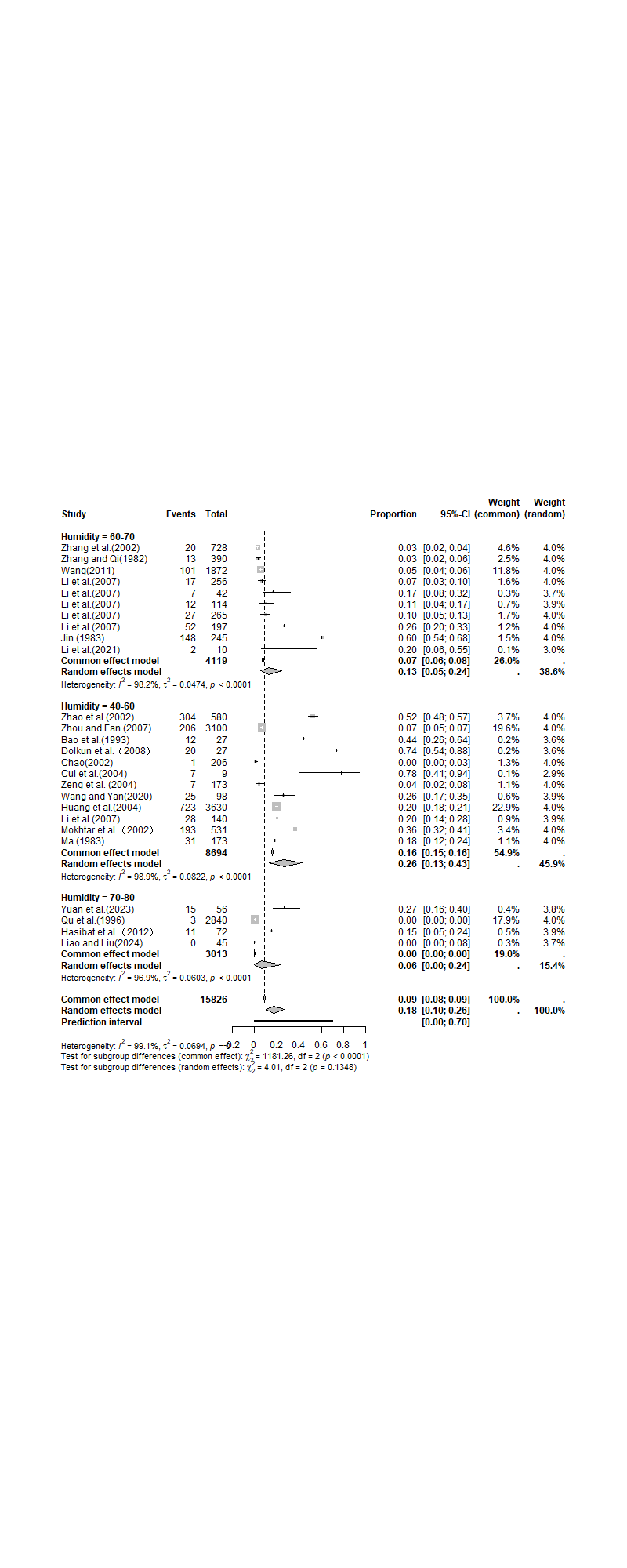


**FIGURE S28. Forest plot of the humidity subgroup.**

**FIGURE S29. Funnel plot with pseudo 95% confidence limit intervals for the examination of publication bias in the average annual temperature subgroup.**


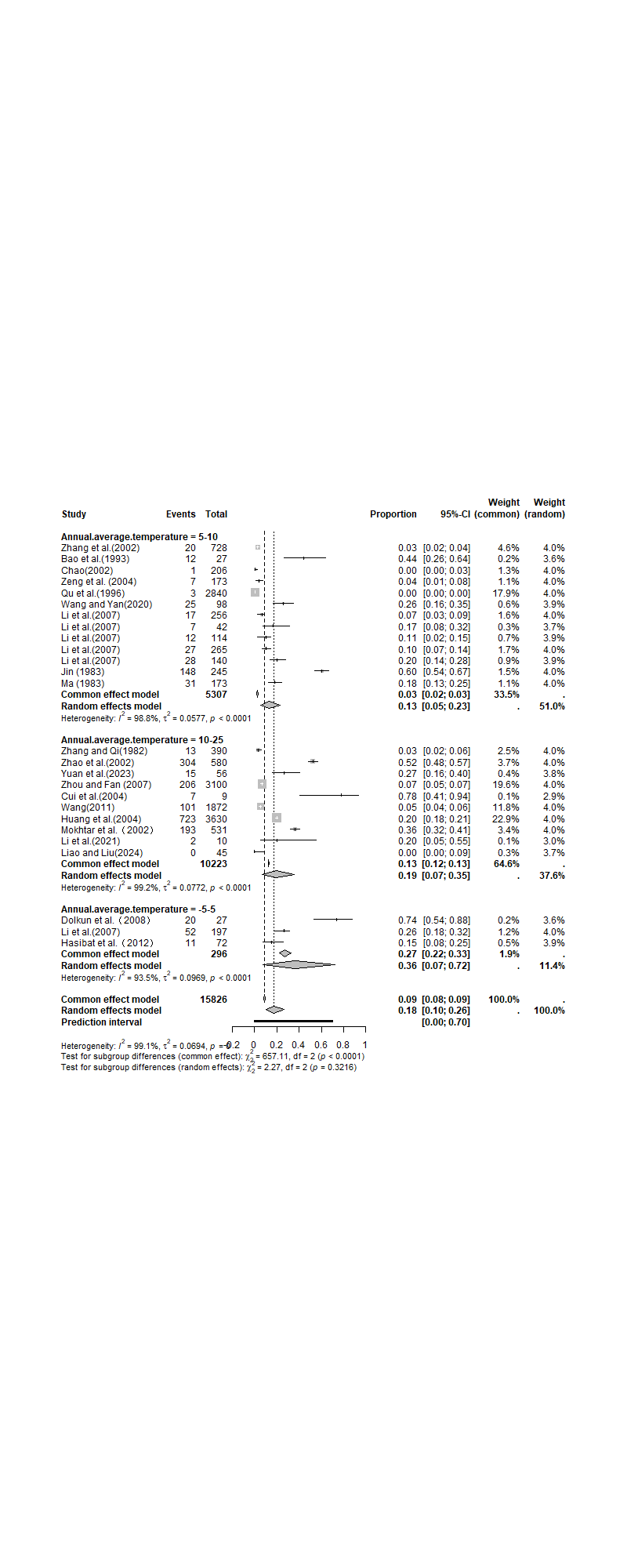


**FIGURE S30. Forest plot of the average annual temperature subgroup.**

**FIGURE S31. Funnel plot with pseudo 95% confidence limit intervals for the examination of publication bias in the climate subgroup.**


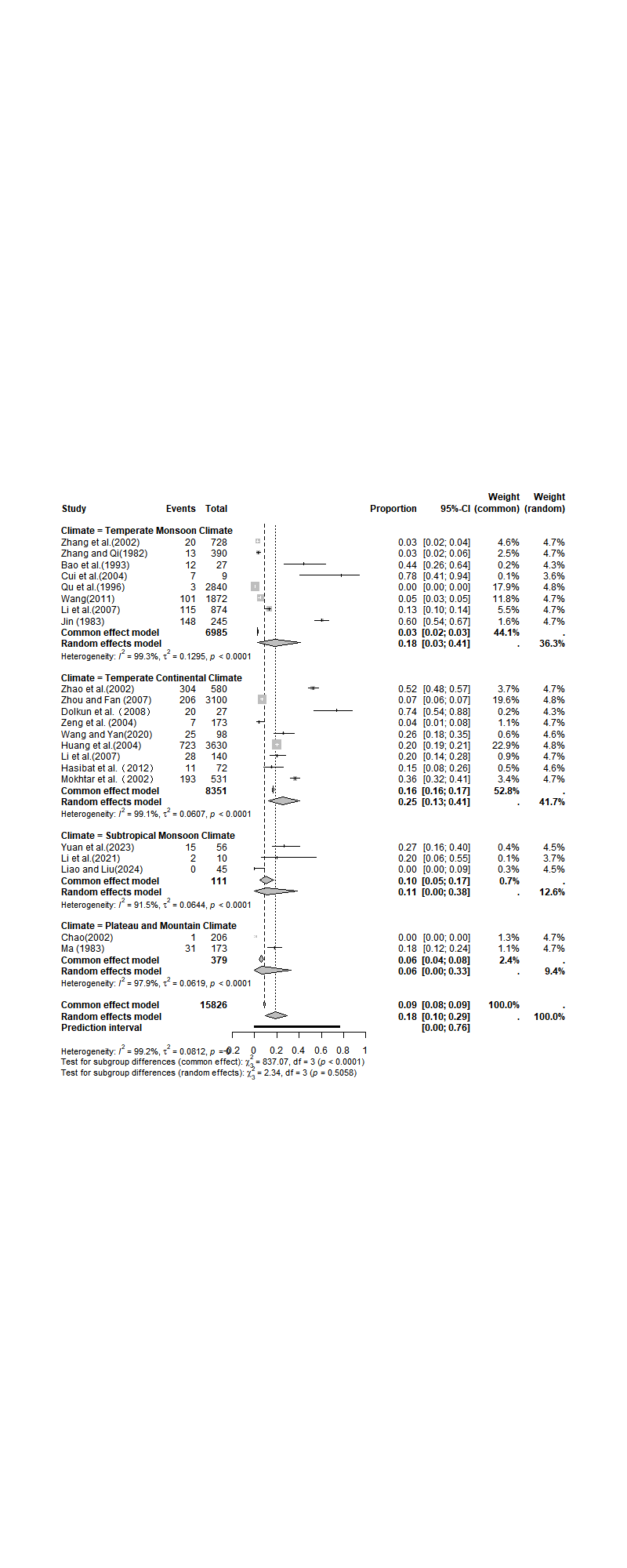


**FIGURE S32. Forest plot of the climate subgroup.**
